# Supplementary material for: Highly multiplexed 3D profiling of cell states and immune niches in human tumors
Source: Nat Methods. 2025 Sep 29;22(10):2180–93. doi: 10.1038/s41592-025-02824-x (PMC12510885; doi:10.1038/s41592-025-02824-x)
Supplement: Supplementary file 1 — Supplementary Figs. 1–21 and Note 1. [file 41592_2025_2824_MOESM1_ESM.pdf]

nature methods

Supplementary information https://doi.org/10.1038/s41592-025-02824-x

Highly multiplexed 3D profiling of cell states and immune niches in human tumors

In the format provided by the authors and unedited

Contents

**Title**	**Pg. no.**
Supplementary Figure 1. Z-projection of full dataset for invasive melanoma (vertical growth phase; VGP) region for tissue section LSP13626.	2
Supplementary Figure 2. Z-projection of full dataset for melanoma in situ (MIS) region for tissue section LSP13626.	3
Supplementary Figure 3. Z-projection of full dataset for invasive melanoma (vertical growth phase; VGP) region for tissue section LSP13625.	4
Supplementary Figure 4. Z-projection of full dataset for melanoma in situ (MIS) region for tissue section LSP13625.	5
Supplementary Figure 5. Z-projection of full dataset for metastatic melanoma (tissue section LSP22409).	6
Supplementary Figure 6. Z-projection of full dataset for lung metastasis (tissue section LSP22408).	7
Supplementary Figure 7. Z-projection of full dataset for glioblastoma (tissue section LSP17378).	8
Supplementary Figure 8. Z-projection of full dataset for serous tubal intraepithelial carcinoma (STIC), region TR3 (tissue section LSP18251).	9
Supplementary Figure 9. Z-projection of full dataset for serous tubal intraepithelial carcinoma (STIC), region TR4 (tissue section LSP18251).	10
Supplementary Figure 10. Z-projection of full dataset for serous tubal intraepithelial carcinoma (STIC), region TR5 (tissue section LSP18251).	11
Supplementary Figure 11. Z-projection of full dataset of tonsil (tissue section LSP13357).	12
Supplementary Figure 12. Tissue processing strategies to improve tissue adherence for fragile samples.	13
Supplementary Figure 13. Flowchart used for cell type calling in melanoma.	14
Supplementary Figure 14. Z-projection of cropped region from Dataset3-LSP22409 metastatic melanoma (from Figure 3f).	15
Supplementary Figure 15. Z-projection of cropped region from Dataset1-LSP13626 melanoma in-situ (from Figure 6m).	16
Supplementary Figure 16. Antibody penetration in tissues of different thicknesses.	17
Supplementary Figure 17: Timelapse of antibody penetration.	18
Supplementary Figure 18. Comparison of antibody penetration with different secondary antibodies against a MART1 primary conjugate.	19
Supplementary Figure 19. Orthogonal views comparing penetration of different antibodies in the same 35-micron thick melanoma tissue section (Dataset 2 – LSP13625).	20
Supplementary Figure 20. Antibody penetration comparison of PCNA conjugated with Alexafluor 488 and Alexafluor 750.	21
Supplementary Figure 21. Antibody penetration comparison of αSMA conjugated with Alexafluor 488 (green) and Alexafluor 750 (magenta).	22
Supplementary Note 1	23

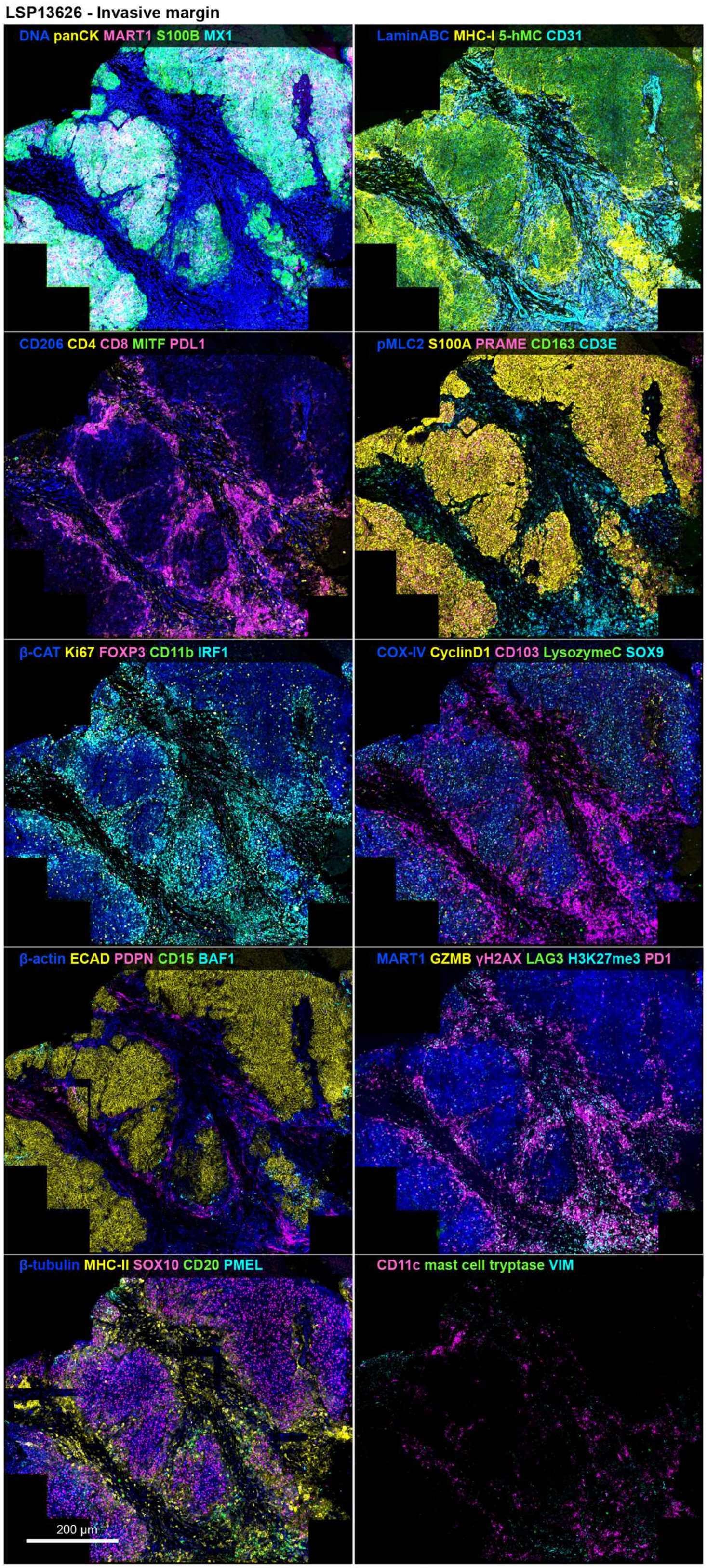

Supplementary Figure 1. Z-projection of full dataset for invasive melanoma (vertical growth phase; VGP) region for tissue section LSP13626. Imaged with 40x/1.3NA oil immersion objective lens on Zeiss LSM980 confocal microscope; sampled at 140nm (x,y) and 280 (z) over a 35-micron thick tissue specimen. See **Supplementary Table 1** for patient info and Minerva story. See **Supplementary Table 3** for marker panel.

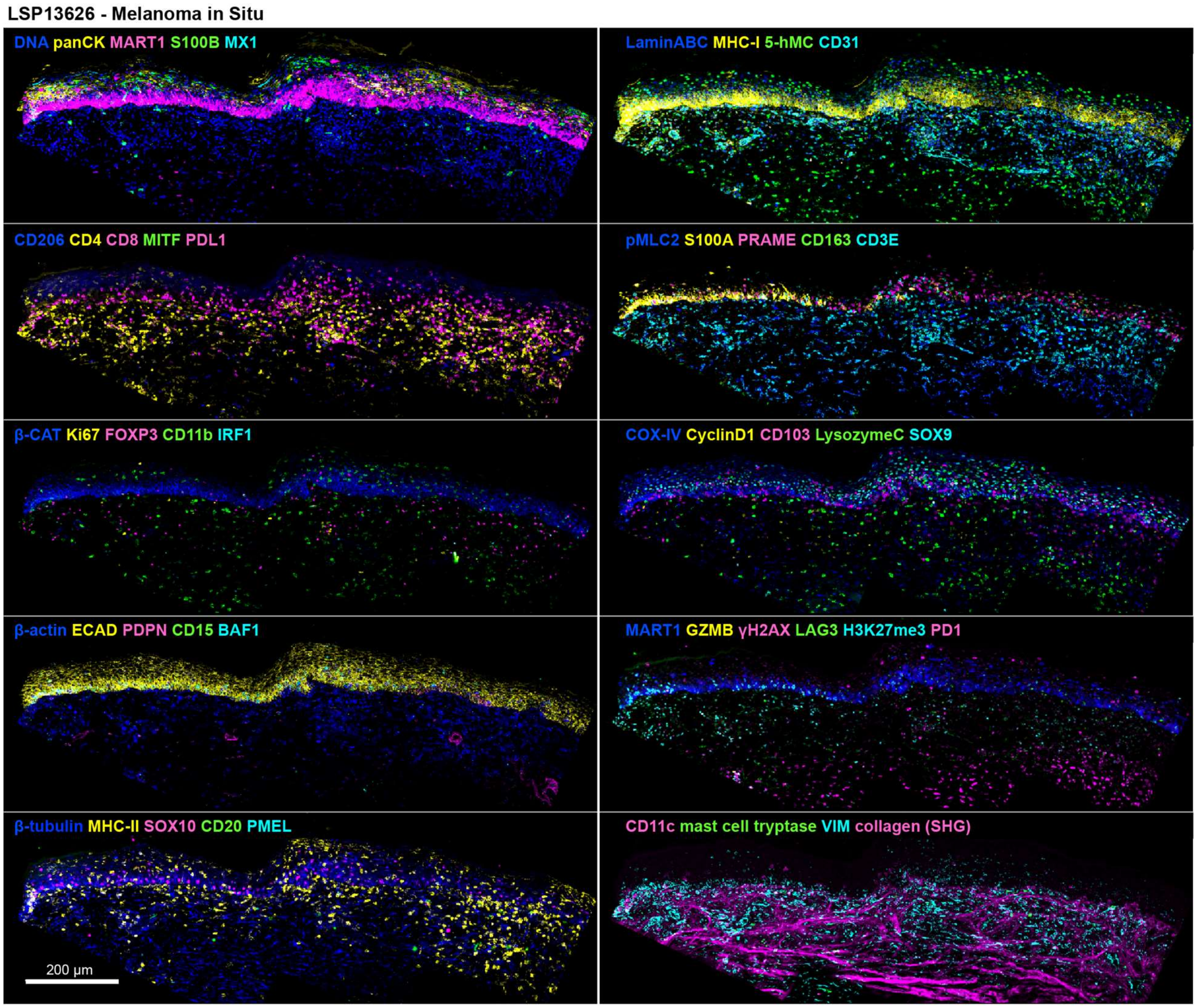

Supplementary Figure 2. Z-projection of full dataset for melanoma in situ (MIS) region for tissue section LSP13626. Imaged with 40x/1.3NA oil immersion objective lens on Zeiss LSM980 confocal microscope; sampled at 140nm (x,y) and 280 (z) over a 35-micron thick tissue specimen. See **Supplementary Table 1** for patient info and Minerva story. See **Supplementary Table 3** for marker panel.

LSP13625- invasive margin

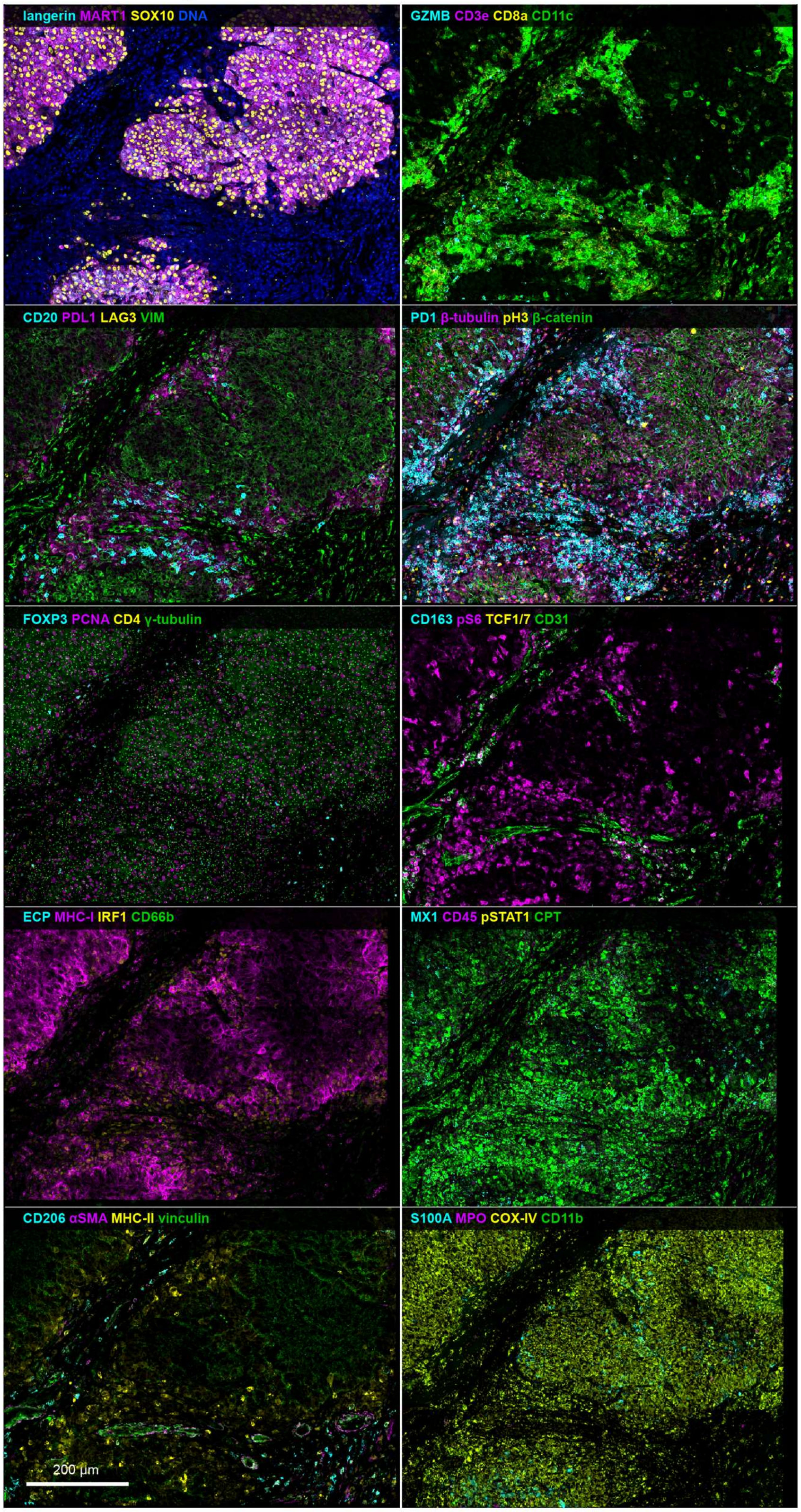

Supplementary Figure 3. Z-projection of full dataset for invasive melanoma (vertical growth phase; VGP) region for tissue section LSP13625. Imaged with 40x/1.3NA oil immersion objective lens on Zeiss LSM980 confocal microscope; sampled at 140nm (x,y) and 280 (z) over a 35-micron thick tissue specimen. See **Supplementary Table 1** for patient info and Minerva story. See **Supplementary Table 4** for marker panel.

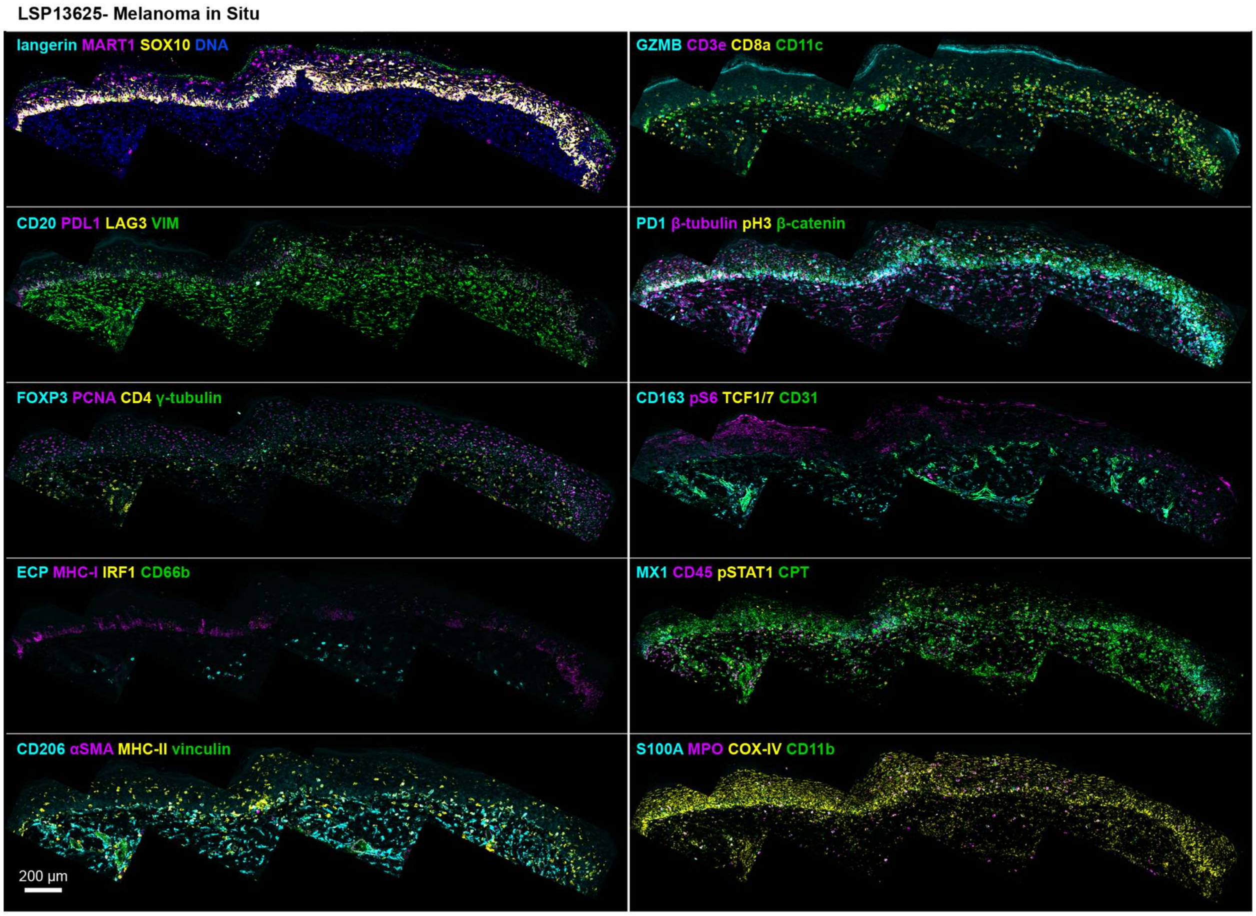

Supplementary Figure 4. Z-projection of full dataset for melanoma in situ (MIS) region for tissue section LSP13625. Imaged with 40x/1.3NA oil immersion objective lens on Zeiss LSM980 confocal microscope; sampled at 140nm (x,y) and 280 (z) over a 35-micron thick tissue specimen. See **Supplementary Table 1** for patient info and Minerva story. See **Supplementary Table 4** for marker panel.

LSP22409 - metastatic melanoma

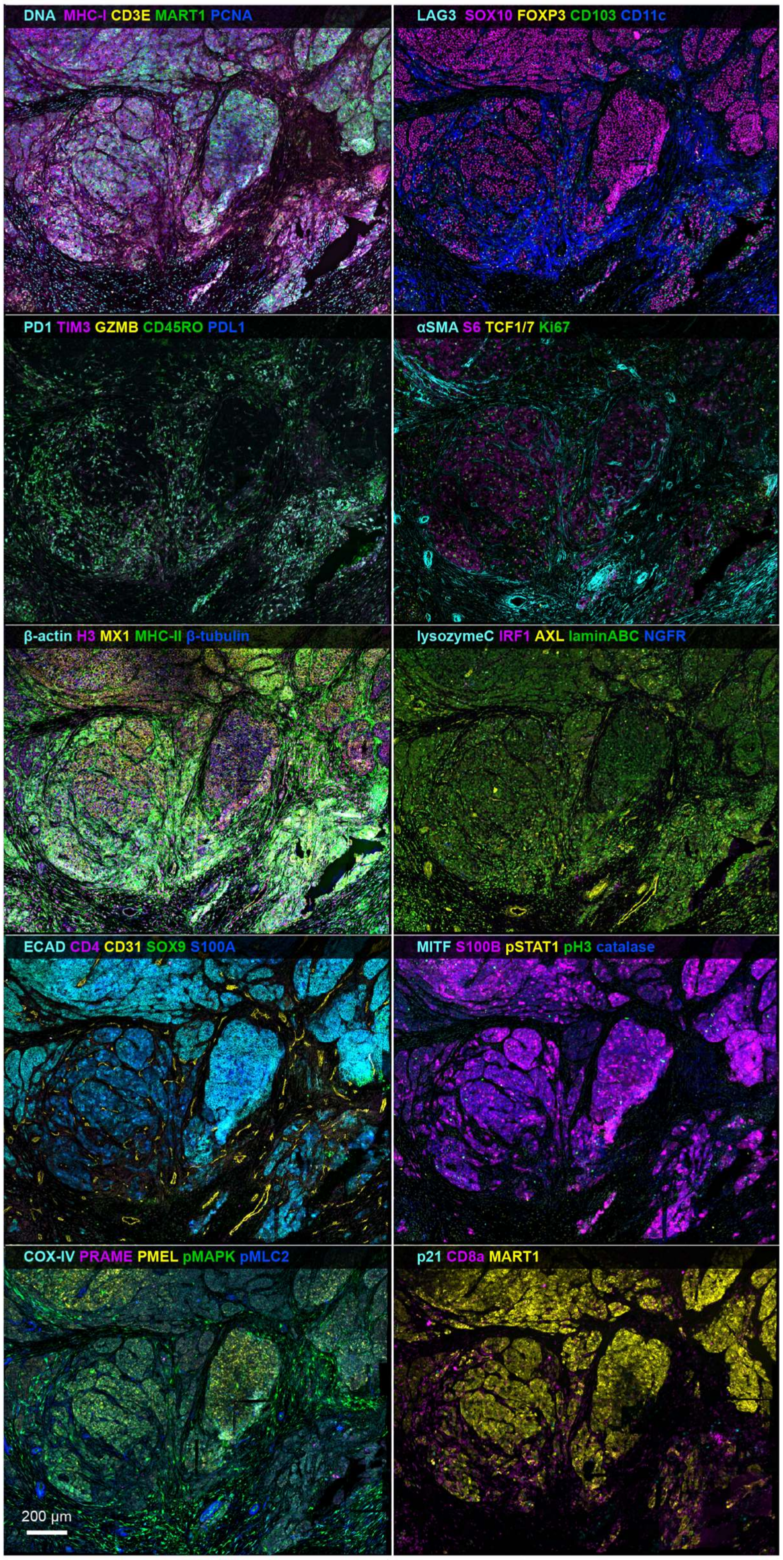

Supplementary Figure 5. Z-projection of full dataset for metastatic melanoma (tissue section LSP22409). Imaged with 40x/1.3NA oil immersion objective lens on Zeiss LSM980 confocal microscope; sampled at 140nm (x,y) and 280 (z) over a 25-micron thick tissue specimen. See **Supplementary Table 1** for patient info and Minerva story. See **Supplementary Table 5** for marker panel.

Lung metastasis - LSP22408

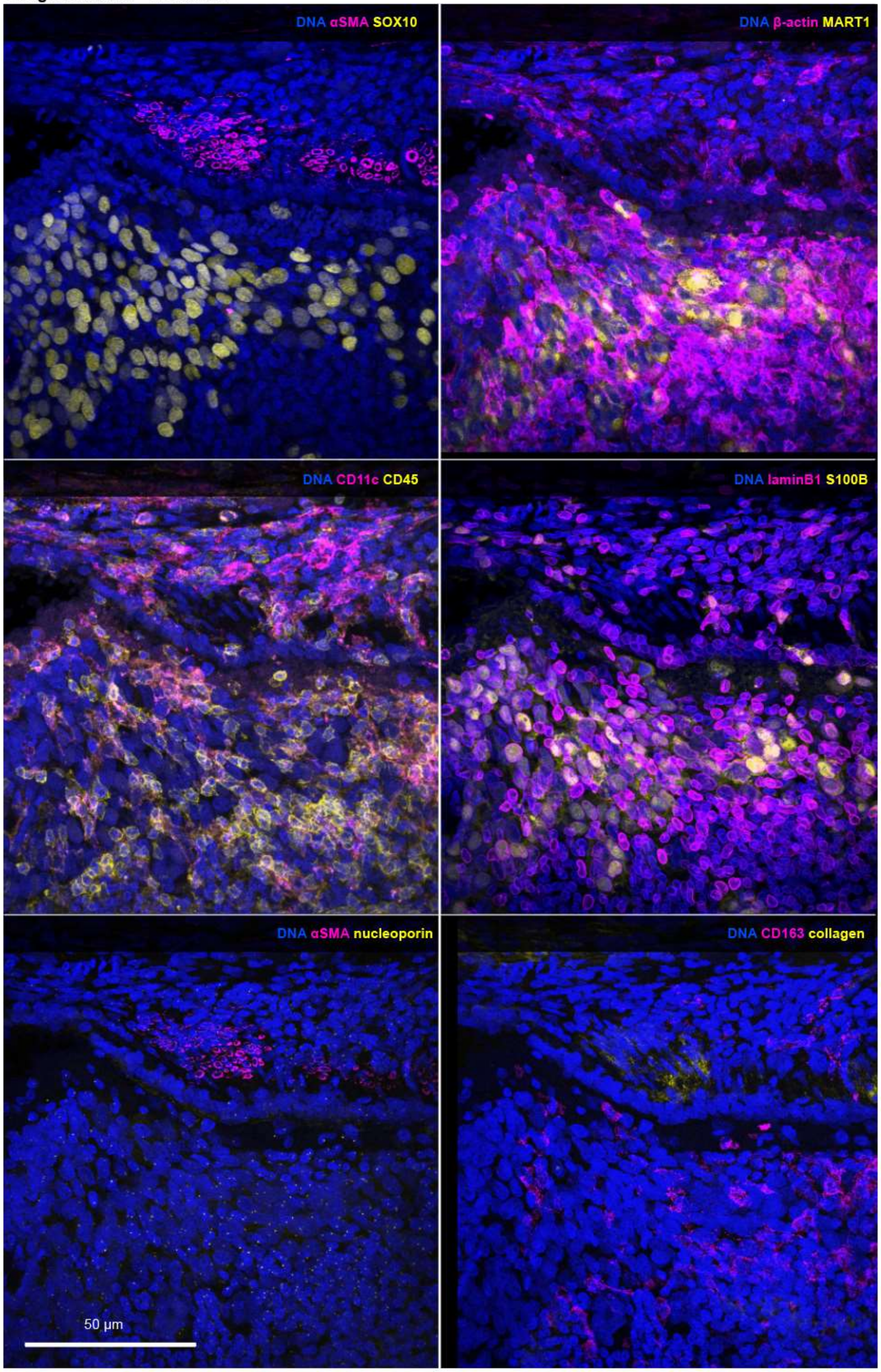

Supplementary Figure 6. Z-projection of full dataset for lung metastasis (tissue section LSP22408). Imaged with 40x/1.25NA silicone oil objective lens on Olympus FV1200 confocal microscope; sampled at 310nm (x,y) and 540 (z) over a 40-micron thick tissue specimen. See **Supplementary Table 1** for patient info and Minerva story. See **Supplementary Table 6** for marker panel.

Glioblastoma - LSP17378

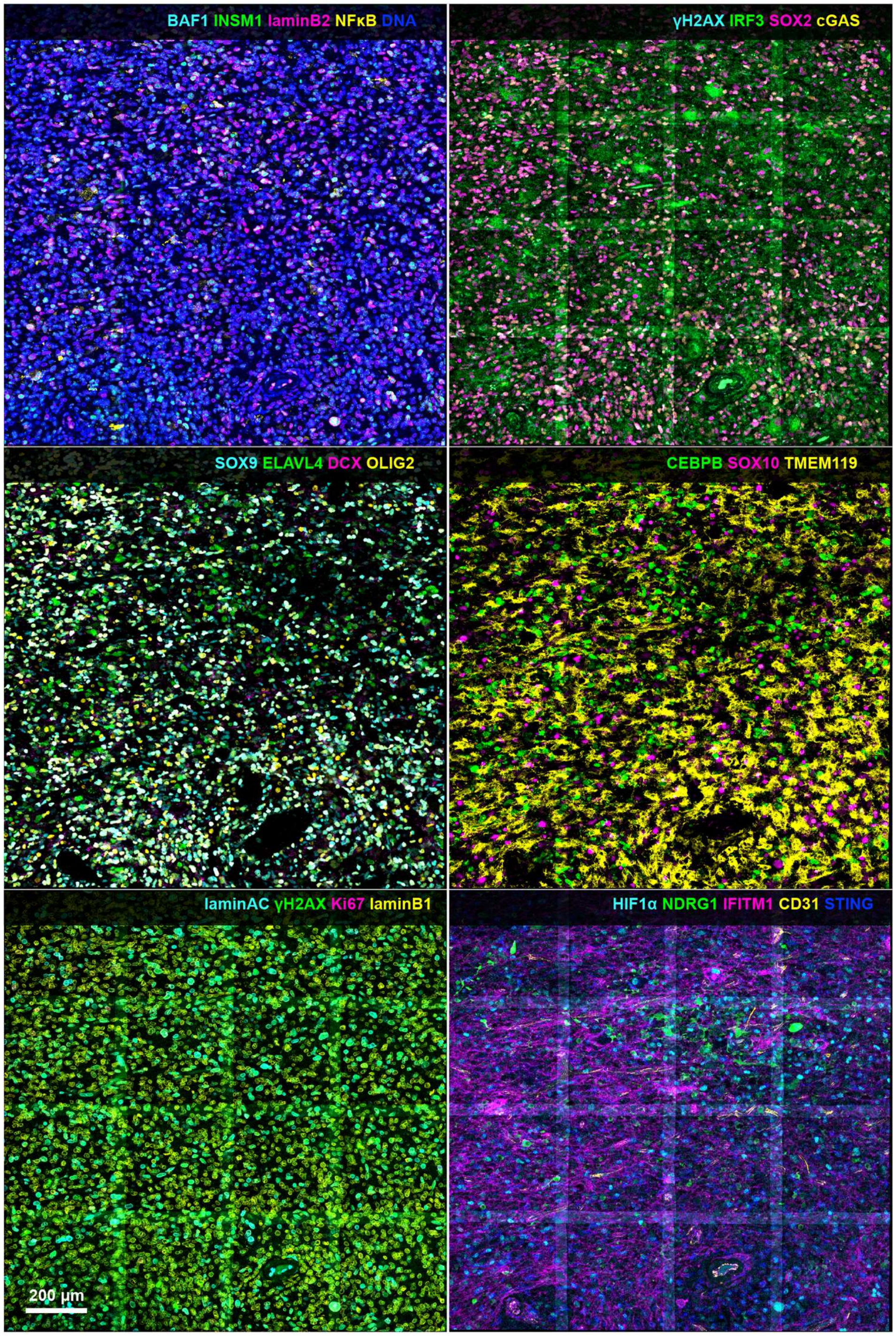

Supplementary Figure 7. Z-projection of full dataset for glioblastoma (tissue section LSP17378). Imaged with 40x/1.3NA objective lens on Zeiss LSM980 confocal microscope; sampled at 140nm (x,y) and 280 (z) over a 20-micron thick tissue specimen. See **Supplementary Table 1** for patient info and Minerva story. See **Supplementary Table 7** for marker panel.

Serous Tubal Intraepithelial Carcinoma (STIC) - region TR3 - LSP18251

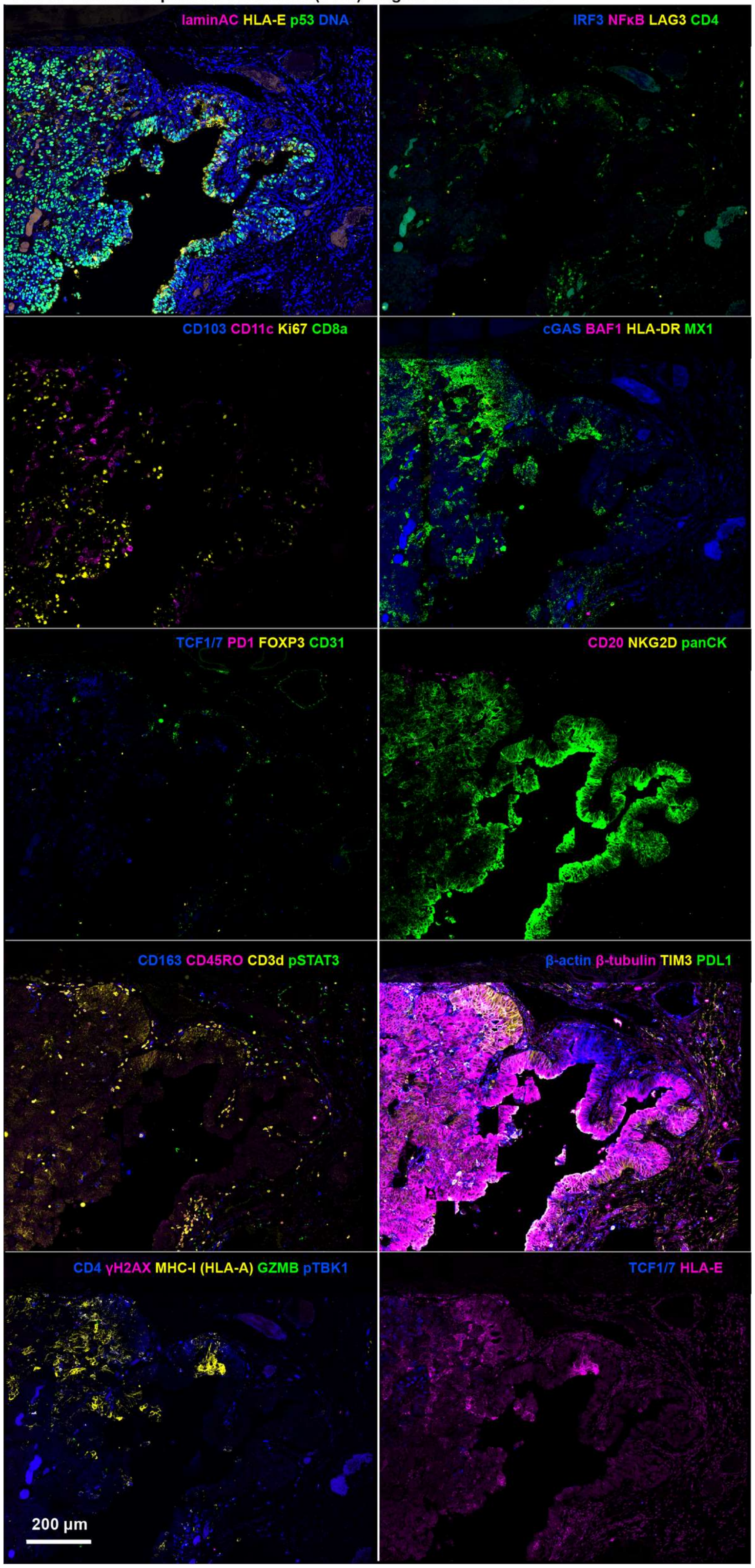

Supplementary Figure 8. Z-projection of full dataset for serous tubal intraepithelial carcinoma (STIC), region TR3 (tissue section LSP18251). Imaged with 40x/1.3NA oil immersion objective lens on Zeiss LSM980 confocal microscope; sampled at 140nm (x,y) and 280 (z) over a 20-micron thick tissue specimen. See **Supplementary Table 1** for patient info and Minerva story. See **Supplementary Table 8** for marker panel.

Serous Tubal Intraepithelial Carcinoma (STIC) - region TR4 - LSP18251

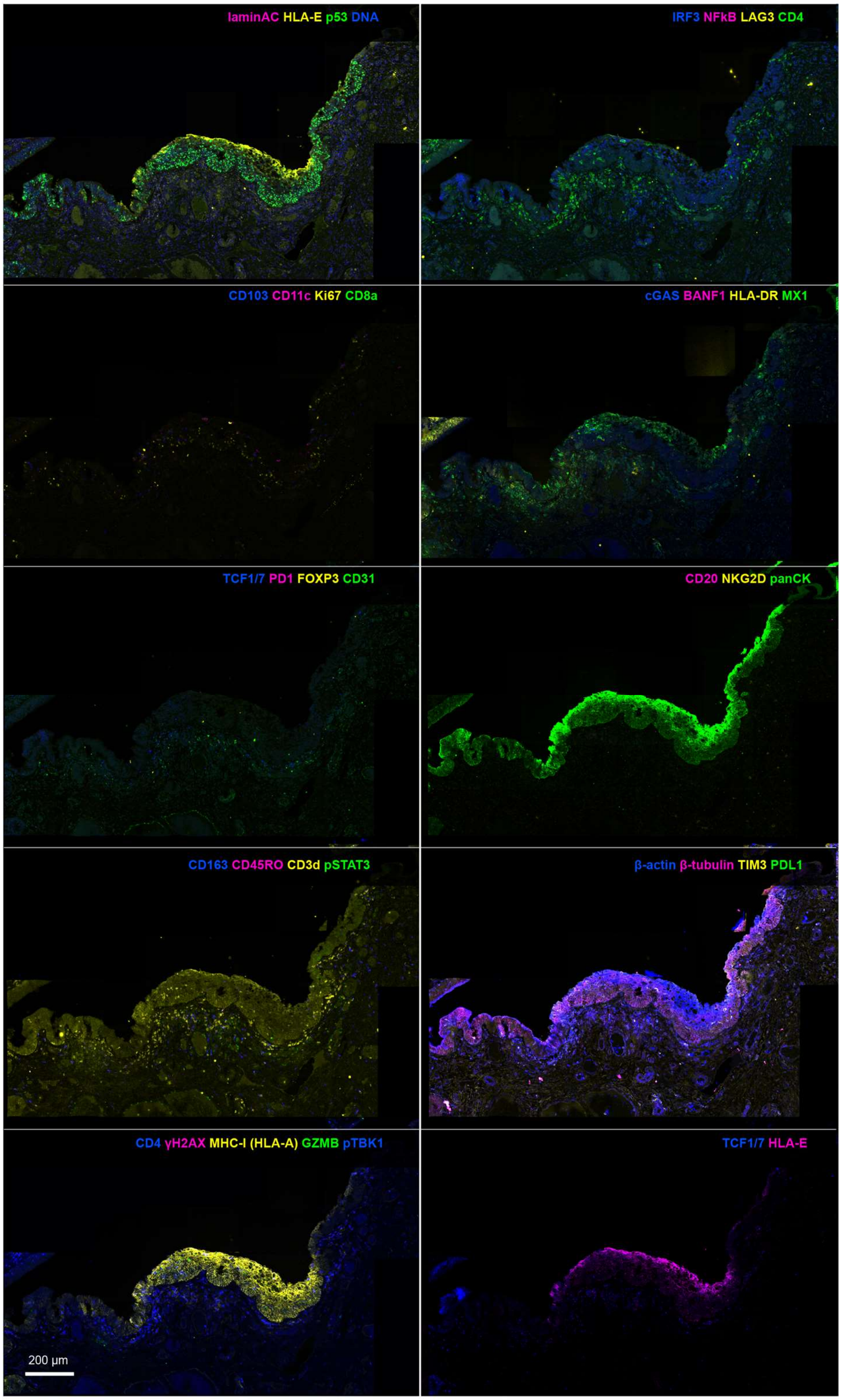

Supplementary Figure 9. Z-projection of full dataset for serous tubal intraepithelial carcinoma (STIC), region TR4 (tissue section LSP18251). Imaged with 40x/1.3NA oil immersion objective lens on Zeiss LSM980 confocal microscope; sampled at 140nm (x,y) and 280 (z) over a 20-micron thick tissue specimen. See **Supplementary Table 1** for patient info and Minerva story. See **Supplementary Table 8** for marker panel.

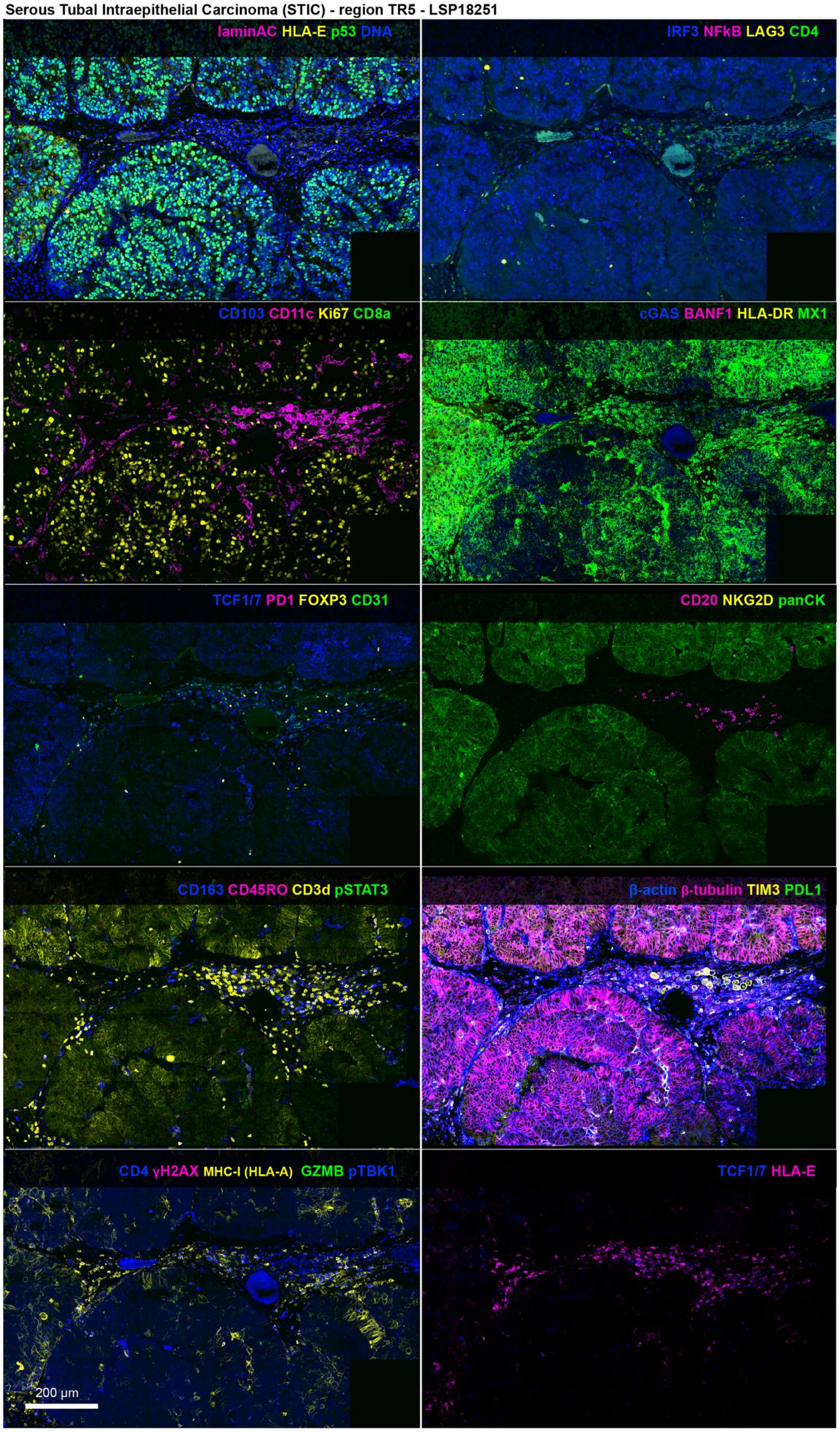

Supplementary Figure 10. Z-projection of full dataset for serous tubal intraepithelial carcinoma (STIC), region TR5 (tissue section LSP18251). Imaged with 40x/1.3NA oil immersion objective lens on Zeiss LSM980 confocal microscope; sampled at 140nm (x,y) and 280 (z) over a 20-micron thick tissue specimen. See **Supplementary Table 1** for patient info and Minerva story. See **Supplementary Table 8** for marker panel.

Tonsil - LSP13357

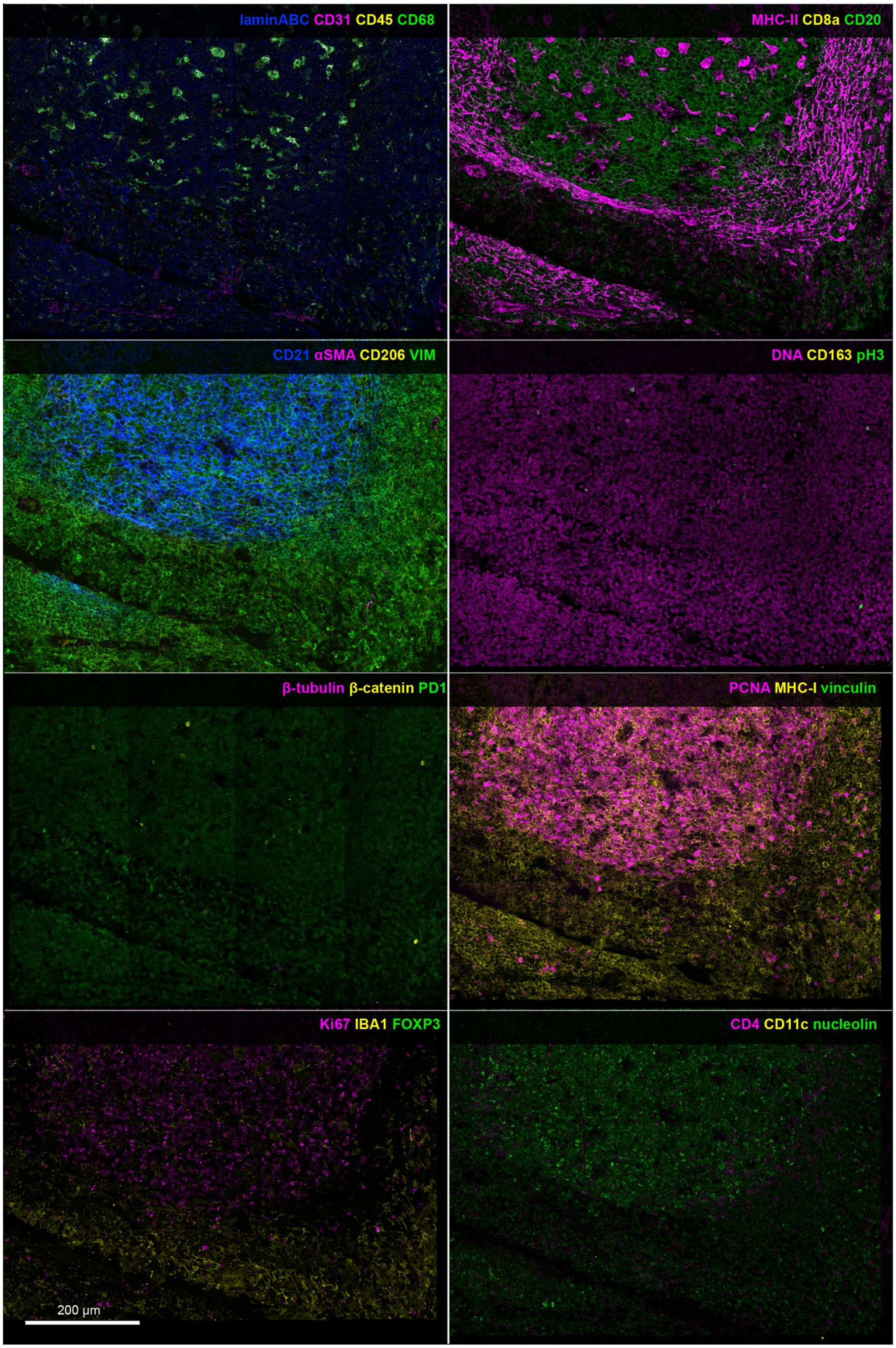

Supplementary Figure 11. Z-projection of full dataset of tonsil (tissue section LSP13357). Imaged with 40x/1.2NA water immersion objective lens on Zeiss LSM980 confocal microscope; sampled at 140nm (x,y) and 280 (z) over a 20-micron thick tissue specimen. See **Supplementary Table 1** for patient info and Minerva story. See **Supplementary Table 9** for marker panel.

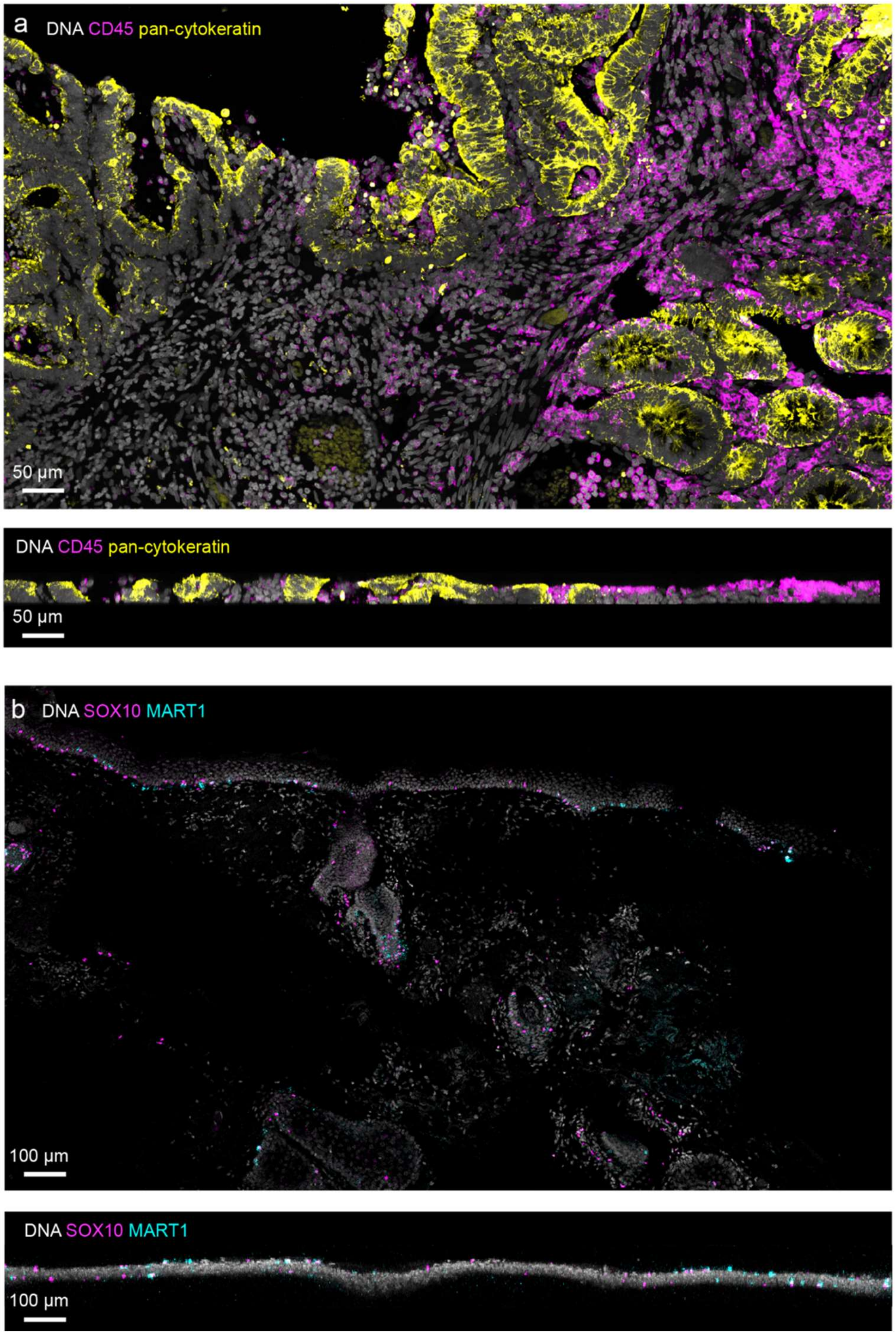

Supplementary Figure 12. Tissue processing strategies to improve tissue adherence for fragile samples. a, XY (top) and XZ (bottom) projection views of a 35-micron thick colorectal cancer sample that survived more than 6 cycles of CyCIF with the set up from **Extended Data Figure 1b** and with Matrigel coated over the sample. XZ projection shows that the tissue tolerated multiple rounds of bleaching and adhered to the coverslip along the length of the tissue. **b,** XY (top) and XZ (bottom) projection views of a fragile melanoma precursor sample (LSP27564 – see **Supplementary Table 10** for marker panel) with the set up from **Extended Data Figure 1b** but with a mesh over the sample. Bottom XZ projection shows that the tissue exhibited significantly poorer adhesion to the coverslip shown by the wavy pattern. Yet the tissue survived 3 cycles of CyCIF as a result of overlaying a protective mesh over the tissue. In contrast, setup from **Extended Data Figure 1a** caused the sample to immediately detach after initial bleaching.

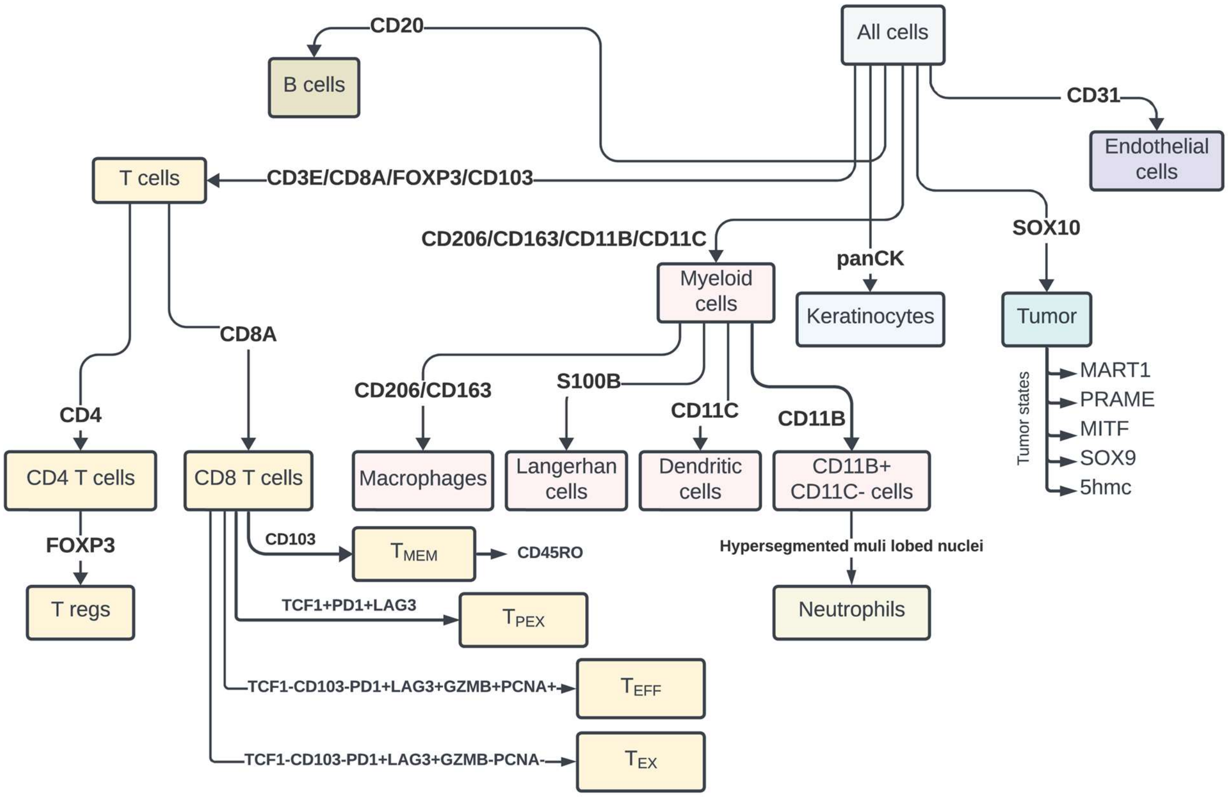

Additional markers that were used to determine cell states and morphologies:

COX-IV	CyclinD1	MX1	pMLC2	β-actin
γH2AX	KI67	IRF1	VIM	β-catenin
	PCNA	pSTAT1	Vinculin	β-tubulin

Supplementary Figure 13. Flowchart used for cell type calling in melanoma. Lines and arrows represent cells that stain positive for the indicated markers. Under tumour cells, specific tumour cell states are shown. Cell states that are not related to a specific marker are shown in the lower box.

Metastatic melanoma (Dataset 3 - LSP22409)

(from Figure 3f from main text)

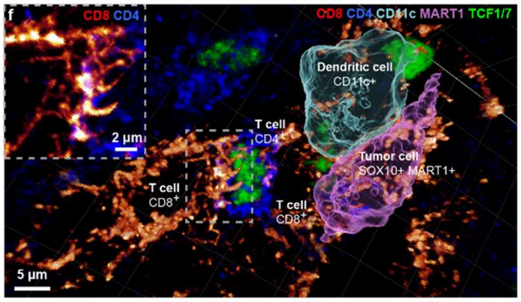

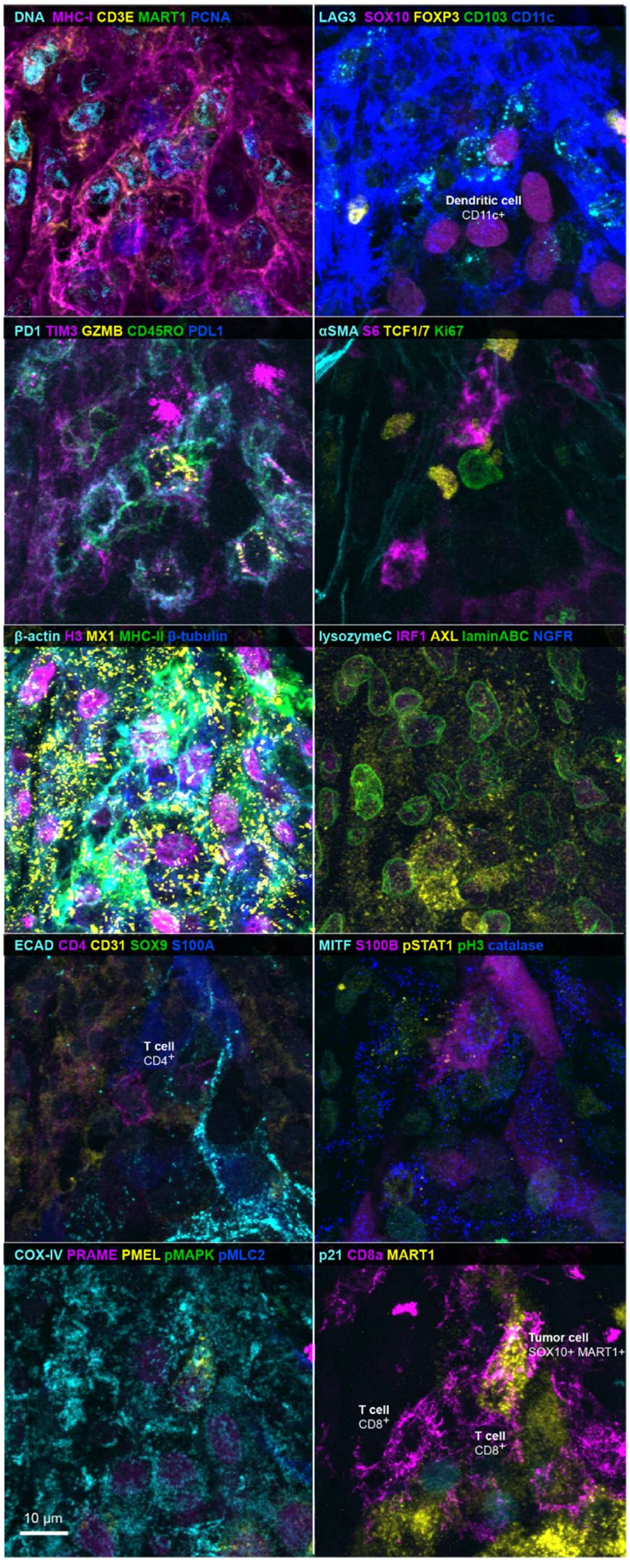

Supplementary Figure 14. Z-projection of cropped region from Dataset3-LSP22409 metastatic melanoma (from Figure 3f). Imaged with 40x/1.3NA oil immersion objective lens on Zeiss LSM980 confocal microscope; sampled at 140nm (x,y) and 280 (z) over a 35-micron thick tissue specimen. See Supplementary Table 1 for patient info and Minerva story. See Supplementary Table 5 for marker panel. Markers represented as colors as indicated.

LSP13626 - Melanoma in-situ

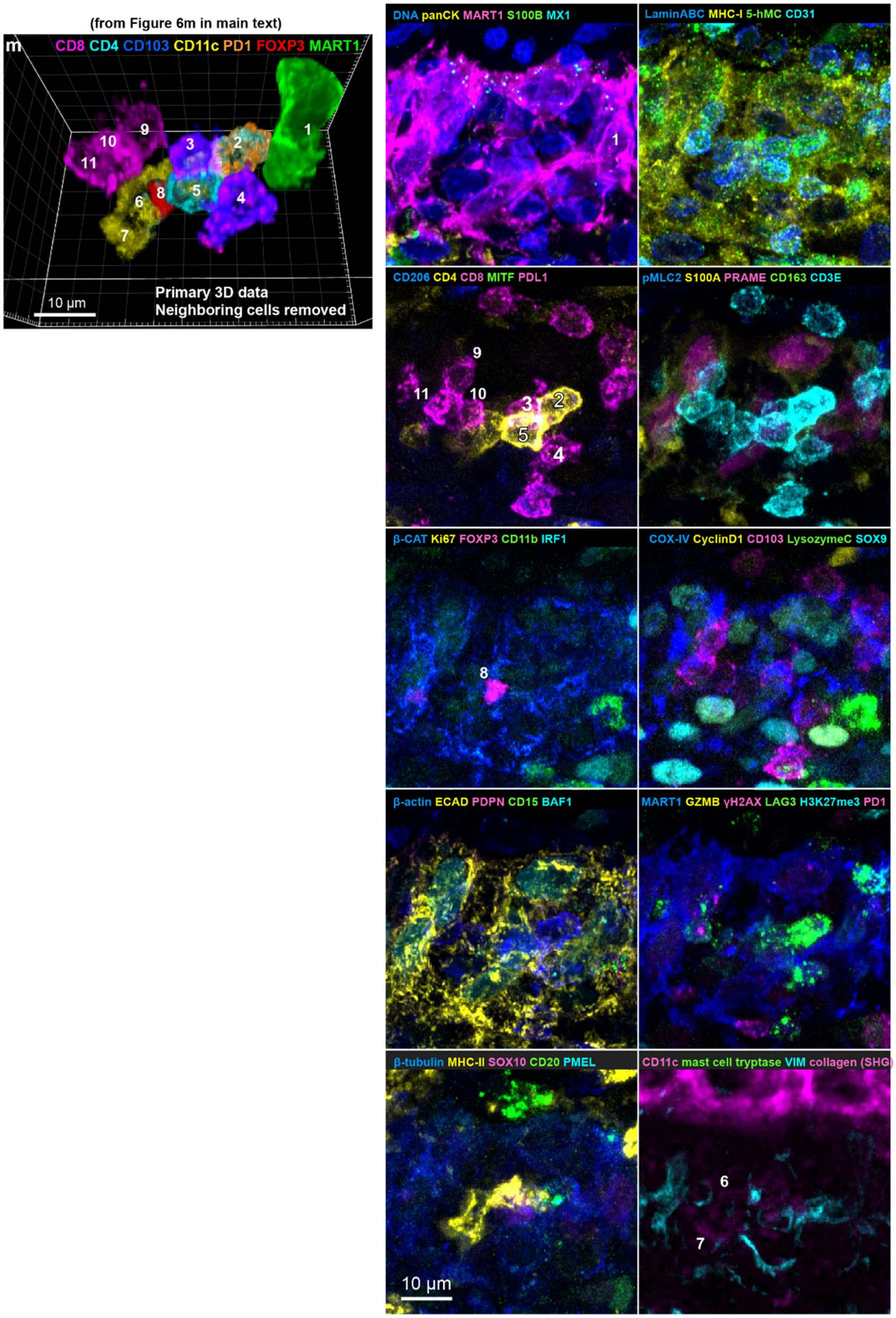

Supplementary Figure 15. Z-projection of cropped region from Dataset1-LSP13626 melanoma in-situ (from Figure 6m). Imaged with 40x/1.3NA oil immersion objective lens on Zeiss LSM980 confocal microscope; sampled at 140nm (x,y) and 280 (z) over a 35-micron thick tissue specimen. See Supplementary Table 1 for patient info and Minerva story. See Supplementary Table 3 for marker panel. Markers represented as colors as indicated.

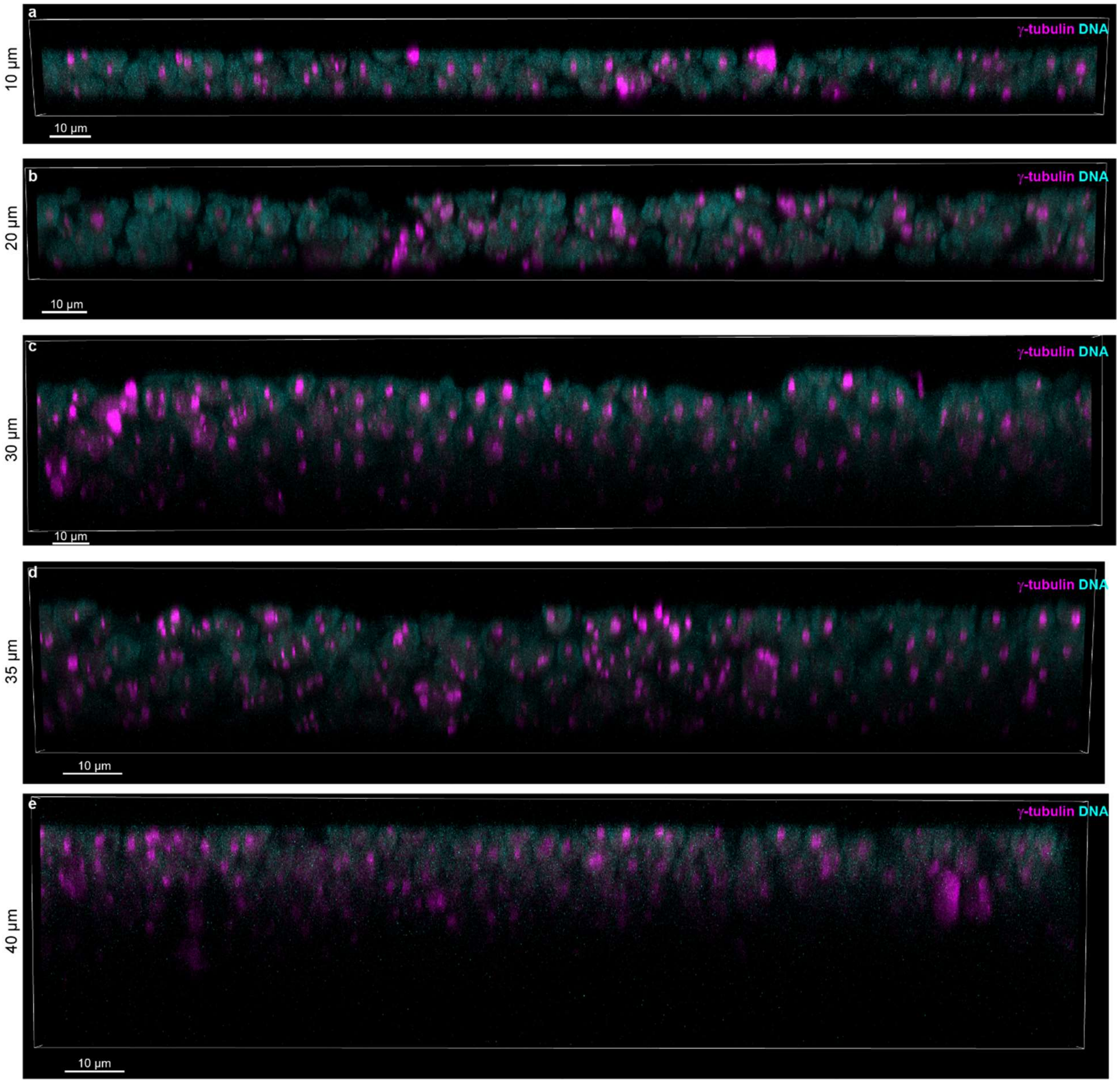

Supplementary Figure 16. Antibody penetration in tissues of different thicknesses. a-e, Orthogonal views of γ-tubulin-Alexafluor 555 staining (magenta) tonsil sections of 10 µm (**a**), 20 µm (**b**), 30 µm (**c**), 35 µm (**d**), and 40 µm (**e**) thicknesses, respectively. γ-tubulin staining can be observed from the top to bottom of the tissue up until 35-micron thick tissue sections. In the 40-micron thick sample, γ-tubulin appears dimmer and significantly distorted at the far side of the sample due to lack of antibody penetration or light attenuation. All tonsil section prepared on glass slides, stained simultaneously at 4 degrees overnight, washed in PBS, and mounted with 70% glycerol. Imaged with 40x/1.2NA water objective lens on Zeiss LSM980 laser scanning confocal microscope sampled at 104nm (x,y) and 230nm (z). DNA, stained with Hoechst, shown in cyan. Scalebar 10 µm.

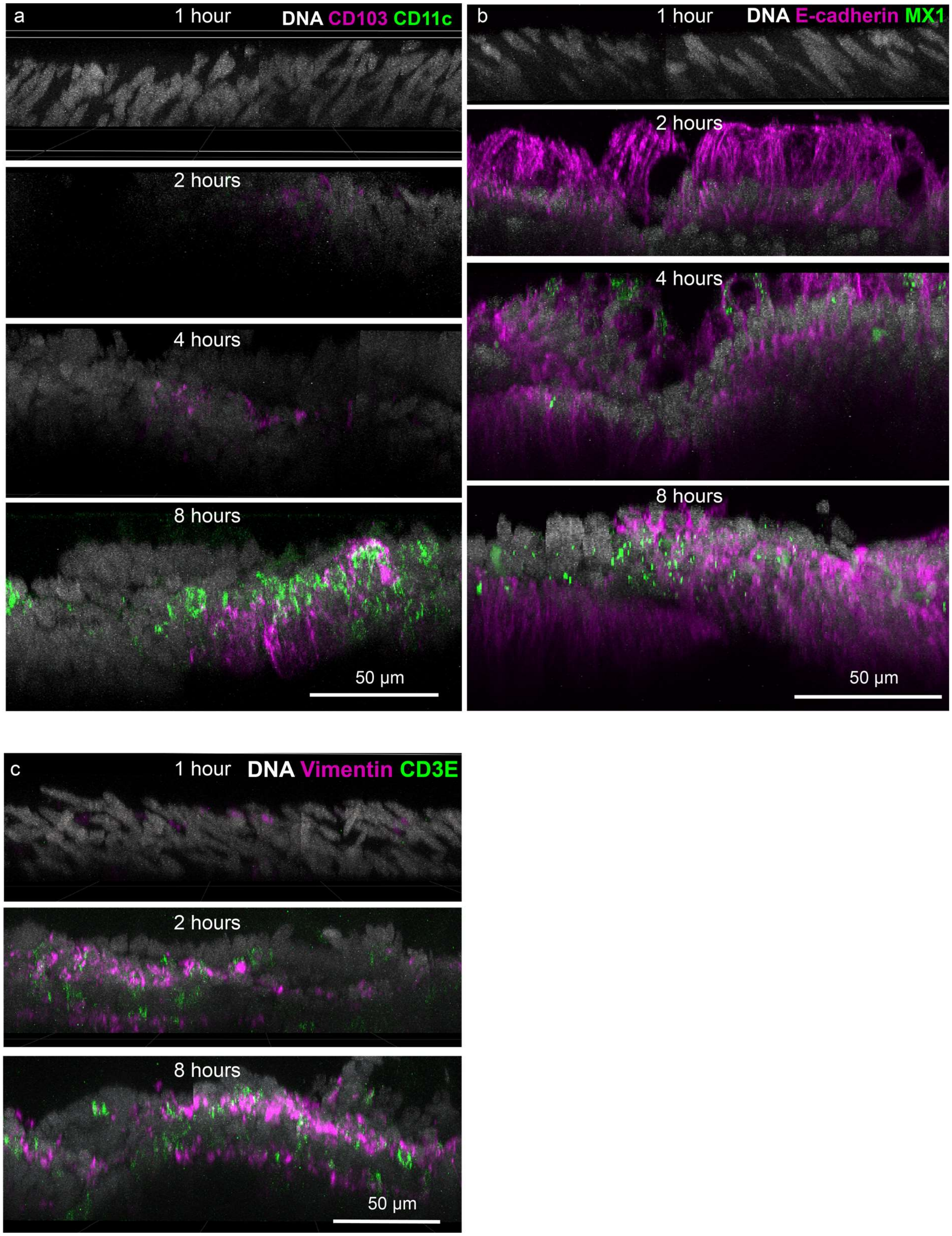

Supplementary Figure 17: Timelapse of antibody penetration. a-c, Orthogonal views of different 35 µm thick colorectal cancer tissue of various groups immune, tumour, and stromal markers imaged after 1, 2, 4 or 8 hours of staining at room temperature and washed in PBS. Staining was not apparent until after 2 hours of incubation. Full antibody penetration was observed after 8 hours of staining at room temperature. Tissue specimens prepared and stained on glass coverslips with vimentin-Alexafluor 750 (magenta), CD3e-Alexafluor 555 (green), Hoechst for DNA (grey) and mounted with 70% glycerol. Imaged with 40x/1.3NA oil objective lens on Zeiss LSM980 laser scanning confocal microscope sampled at 414nm (x,y) and 290nm (z).

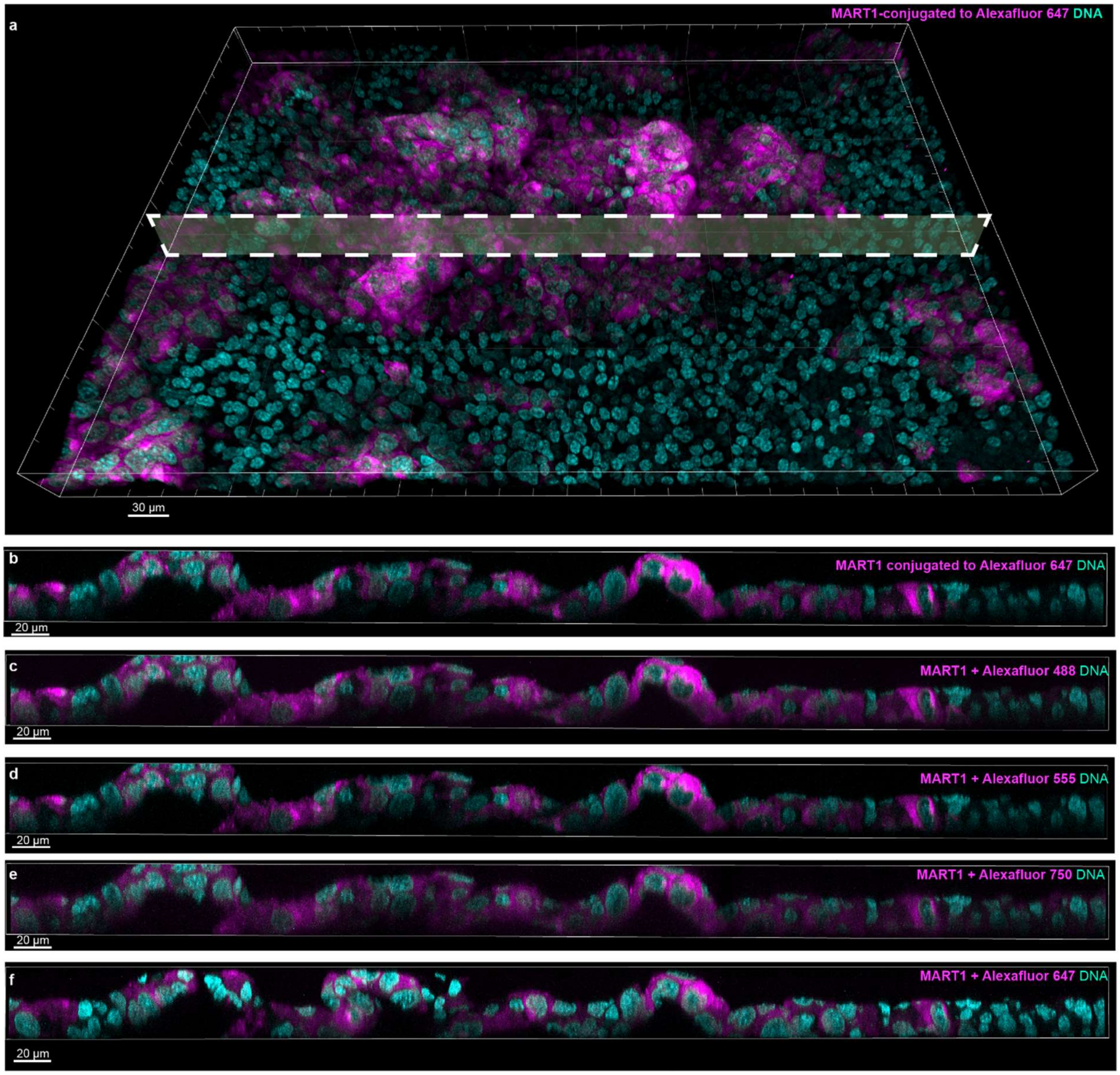

Supplementary Figure 18. Comparison of antibody penetration with different secondary antibodies against a MART1 primary conjugate. a, Volume rendering of primary melanoma, with dashed white rectangle indicating the location of the orthogonal views in **b-f**. Scale bar 30 µm. **b-f,** MART1-conjugated to Alexafluor 647 (**b**) Alexafluor 488 (**c**), Alexafluor 555 (**d**), Alexafluor 750 (**e**), or Alexafluor 647 (**f**). All combinations except MART1 + Alexafluor 647 were stained in the same cycle. MART1 + Alexafluor 647 was stained on the next cycle at the same location and same tissue specimen. Results indicate that, for MART1, fluorophore did not affect staining penetration. The use of primary conjugated vs unconjugated also did not influence staining pattern. Tissue section prepared and stained on glass coverslip at 4 degrees C overnight, washed in PBS, and mounted with 70% glycerol. Imaged with 40x/1.3NA oil objective lens on Zeiss LSM980 laser scanning confocal microscope sampled at 207nm (x,y) and 280nm (z). Scale bars 20 µm.

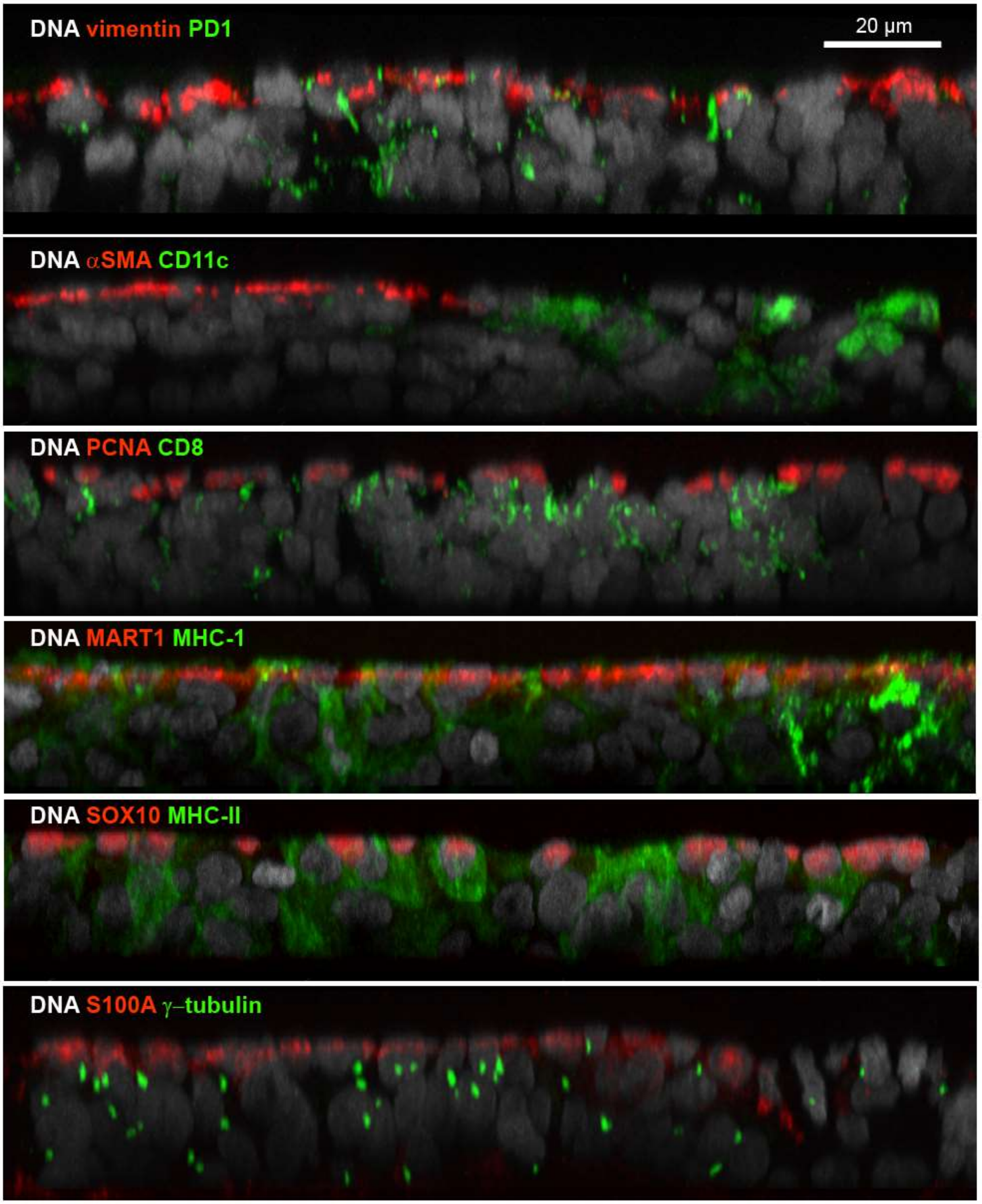

Supplementary Figure 19. Orthogonal views comparing penetration of different antibodies in the same 35-micron thick melanoma tissue section (Dataset 2 – LSP13625). Various antibodies (red) can exhibit poor antibody penetration whereas other antibodies (green) penetrate the full thickness of tissue. Tissue specimen was stained with antibodies as indicated across multiple rounds of CyCIF at 4 degrees C overnight on glass slide, washed in PBS, and mounted with 70% glycerol. Imaged with 40x/1.3NA oil objective lens on Zeiss LSM980 laser scanning confocal microscope sampled at 140nm (x,y) and 280nm (z). DNA stained with Hoechst shown in grey. Scale bar 20 µm.

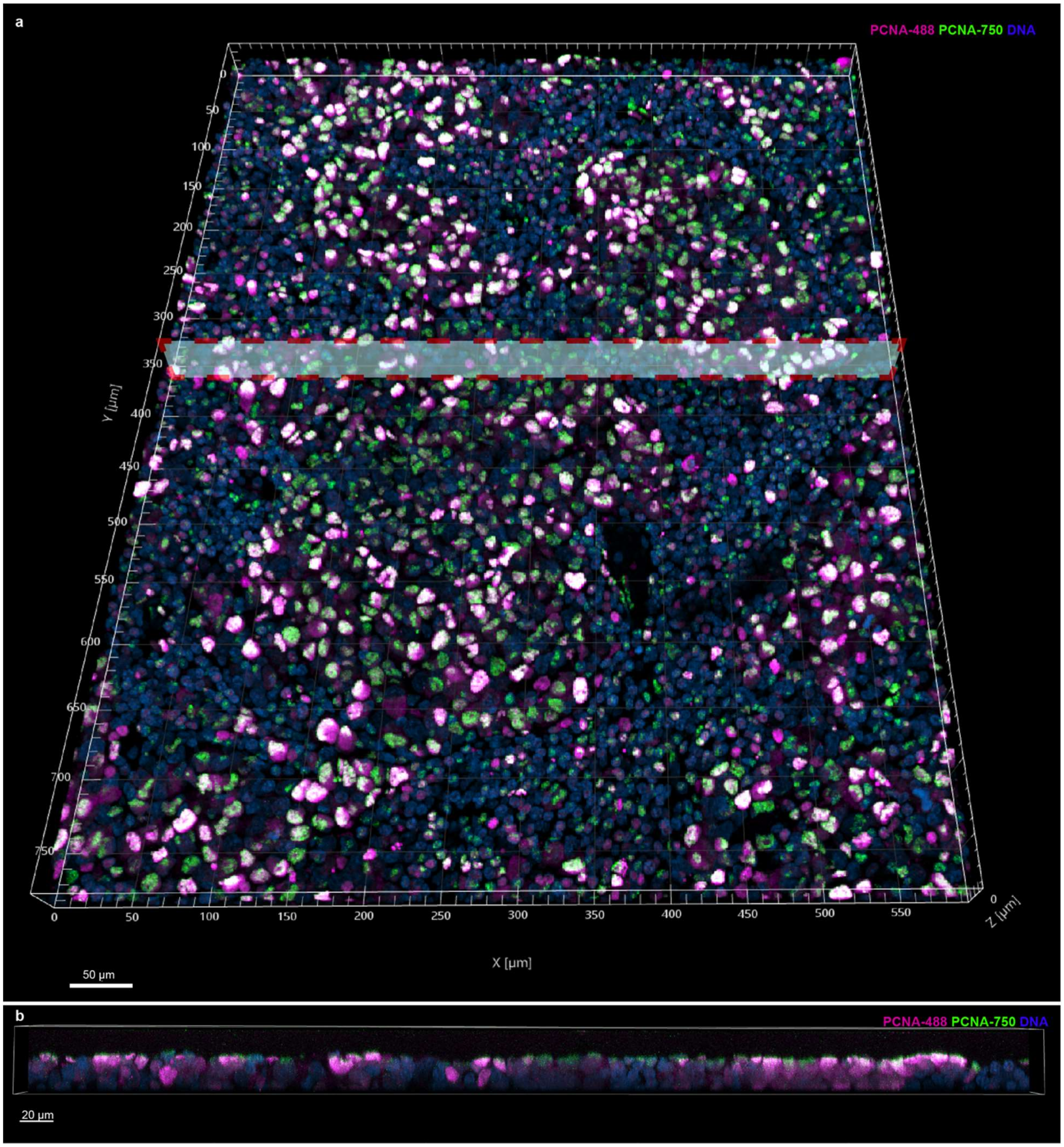

Supplementary Figure 20. Antibody penetration comparison of PCNA conjugated with Alexafluor 488 and Alexafluor 750. a, Volumetric rendering of 35 µm thick primary melanoma. Dashed red rectangle indicates location of orthogonal view in **b**. Scalebar 50 µm. **b** Results show that, in some antibodies such as PCNA, changing fluorophores can affect antibody penetration. For example, PCNA-488 (magenta) penetrates tissue more deeply than PCNA-750 (green). Tissue prepared and stained on glass slide at 4 degrees C overnight, washed in PBS and mounted with 70% glycerol. Imaged with 40x/1.3NA oil objective lens on Zeiss LSM980 laser scanning confocal microscope sampled at 140nm (x,y) and 280nm (z). Scalebar 20 µm.

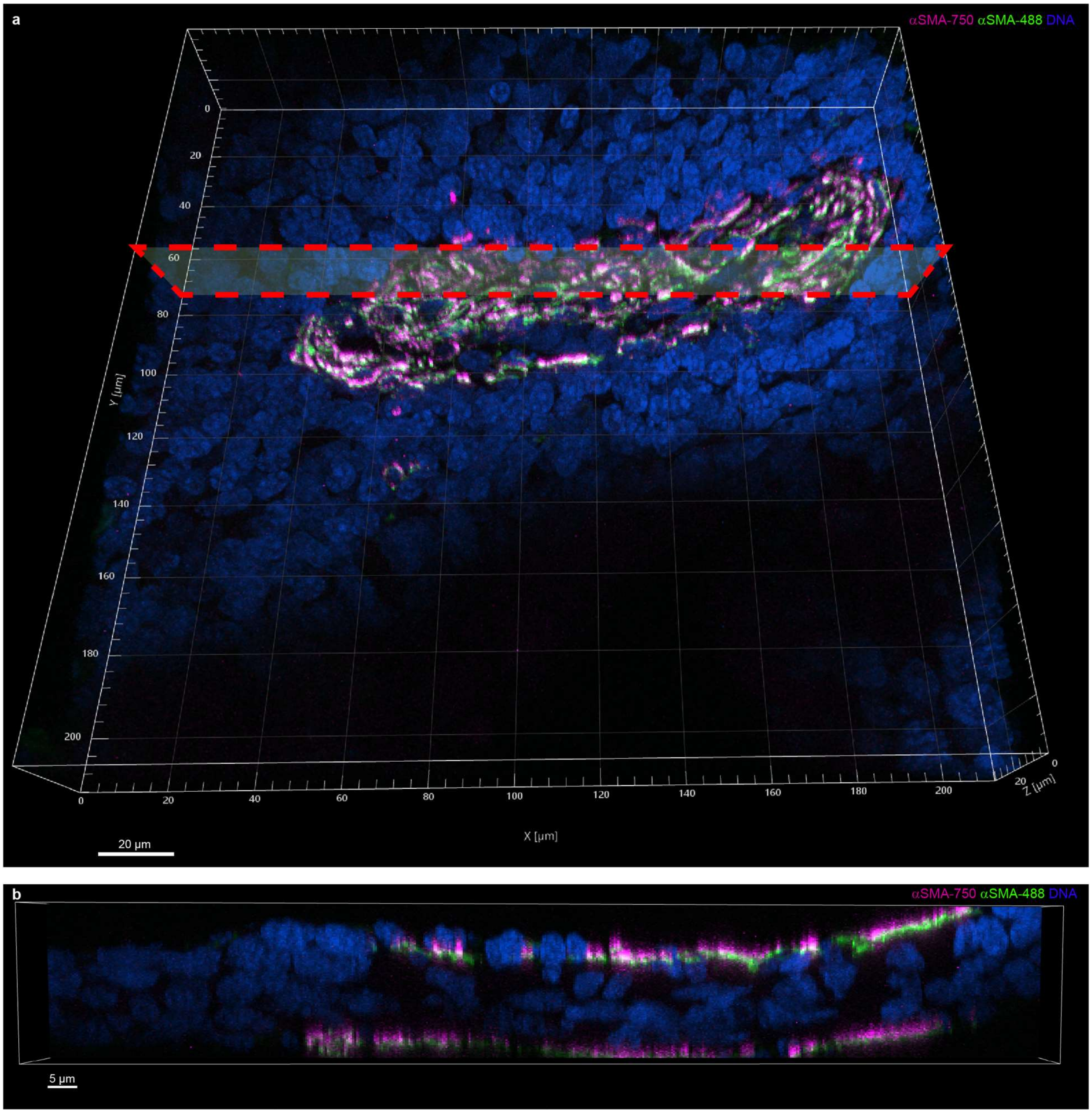

Supplementary Figure 21. Antibody penetration comparison of αSMA conjugated with Alexafluor 488 (green) and Alexafluor 750 (magenta). a, Volumetric rendering of primary melanoma with dashed red rectangle indicating location of orthogonal view in **b**. Scalebar 20 µm. **b,** Cross-sectional view of tissue. Both αSMA antibodies stain only the surface of the tissue regardless of fluorophore conjugated. DNA stained with Hoechst (blue). Tissue prepared and stained on glass slide at 4 degrees C overnight, washed in PBS and mounted with 70% glycerol. Imaged with 40x/1.3NA oil objective lens on Zeiss LSM980 laser scanning confocal microscope sampled at 414nm (x,y) and 300nm (z). Scalebar 5 µm.

SUPPLEMENTARY NOTE 1

APPROACHES TO IMAGING, ANALYZING AND VISUALIZING TISSUES IN 3D

This supplementary note addresses our approach to 3D tissue imaging in the context of contemporary microscopy methods, as well as important technical questions about data analysis and visualization. These issues arose during the review of the current manuscript but length limitations precluded a discussion of them in the main text.

1. Choice of data acquisition approach

A variety of microscopy techniques are available to the research community for the purpose of acquiring high-plex 3D images from different types of specimens (cultured cells, model organisms, and human tissues). These methods each have strengths and weaknesses and this note aims to justify the approach we took in the current paper with a focus on optical imaging.

High-plex optical imaging (>6-10 channels) generally involves cyclic data acquisition. Specimens are stained with 3-8 fluorescently labelled antibodies (and fluorescent dyes such as DAPI and Hoechst 33342), imaged, and then subjected to fluorophore bleaching and/or antibody stripping, followed by another round of staining and imaging; 40-100 plex images can routinely be collected in this way. One of the simplest approaches to creation of 3D datasets is to acquire conventional 2D images from serial sections[1–3] and then assemble them, using image registration, into a 3D volume. Serial section reconstruction is particularly valuable in the setting of multi-omic studies, for example, by alternating protein imaging and spatial transcriptomics on serial tissue sections. However, this approach has several drawbacks. First, it is relatively laborious, and with clinical specimens of varying ages, it requires a high level of expertise to make sure sections are not lost. Second the widefield microscopes (slide scanners) used in 3D reconstruction efforts to date are usually fitted with low numerical aperture (NA) objectives resulting in relatively poor resolution: ~500 nm laterally and roughly the thickness of a section axially (e.g. ~ 5 µm). Moreover, sectioning introduces distortions at the location of the cutting plane.

Multiple methods exist for “true” 3D high-plex imaging. One is to use high resolving power (high NA) objective lenses that collect photons from a small region of the image in the X,Y plane and - importantly - also in Z. In widefield microscopy with high-NA lenses, lateral and axial resolution can be optimized by using deconvolution methods that reassign out-of-focus light back to the focal plane from which they arose (based on knowledge of the point spread function). In confocal microscopy, similar resolution is achieved using a pinhole that rejects out-of-focus light. In both cases, the result is a series of image planes ideally spaced ~250 nm apart each with a lateral resolution of ~200-250 nm (the effective axial resolution in this case is ~500 nm). This is

~3-4 fold smaller in X,Y and 5-fold smaller in Z than a conventional slide scanner (assuming a 0.5-0.8 NA objective). A key point is that high-resolution optical imaging using deconvolution or confocal microscopy is inherently 3D. This is one reason conventional slide-scanners use lower NA objectives: these objectives collect data in focus from more of the specimen along the Z axis, obviating the requirement or multiple image stacks, which improves throughput and reduces file size. Stated another way, in a low resolution 2D image, each pixel samples more of the specimen than in a high resolution 3D image.

We have previously demonstrated the use of widefield deconvolution microscopy with standard cut@5μm specimens,[4,5] but find that the approach becomes less effective as specimens get thicker due to increasing light scatter. In contrast, contemporary laser scanning confocal microscopes such as the Zeiss LSM980 remain effective at rejecting out-of-focus light even with thicker tissue specimens in the presence of high autofluorescence and scatter.

Confocal microscopy can be performed using both spinning disk[6] and laser scanning[7,8] microscopes. We tested both. The latter is slower due to the need to scan the image with a raster, but it removes pinhole crosstalk, provides finer control over the pinhole diameter, and thus, increases the precision of optical sectioning; it also has higher contrast due to superior rejection of out-of-focus light. However, confocal microscopes are relatively inefficient at collecting emitted light because orders of magnitude fewer photons reach the detector in a confocal than a widefield microscope[9] and detectors have lower quantum efficiency than scientific-grade cameras. Additionally, under most confocal imaging conditions, the full sample thickness (at any specific point in X,Y) is fully illuminated along the Z axis regardless of which focal plane is being imaged, contributing to photobleaching. These issues are known to be problematic with live-cell microscopy, but a key conclusion of our research is that they are not major issues in the performance of high-plex imaging of fixed tissue using antibodies and fluorophore (and the use of anti-fade reagents further preserves fluorescent signals).

1.1 Other methods for collecting 3D data: Light Sheet Fluorescence Microscopy.
Over the last few decades a range of 3D imaging has been developed and several of these have potential advantages over confocal microscopy. Light Sheet Fluorescence Microscopy (LSFM) of specimens subjected to tissue clearing[10–14] is an ideal way to image tissue sections as thick as several mm. The most recent systems are capable of subcellular resolution (ca. 0.7 μm laterally). However existing clearing methods are generally incompatible with FFPE tissue[15], and most LSFM imaging to date has been performed on model organisms for which fixation methods other than FFPE are routinely feasible. The total number of fluorophores that can be imaged in a single LSFM cycle is typically 3-5 depending on the optics,[16,17] and a usable implementation of cyclic LSFM has not yet been reported. Specialized methods have been

developed for higher-plex 3D LSFM imaging in thick animal tissues[18,19] or thin 5 µm sections[20]. However, these methods remain difficult to implement with human specimens and they do not achieve the same resolution as thick-section confocal CyCIF. Our experience is that LSFM of tissues is ideal for imaging extended tissue structures such as nerves, blood vessels, and multi-cellular assemblies such as colonic villi or hair follicles. However, the lower resolution of LSFM as compared to confocal microscopy, and the necessity of developing FFPE-compatible clearing protocols, suggest that LSFM is a complement rather than a replacement for the thick-section confocal methods described here.

1.2 Additional 3D imaging modalities including two-photon microscopy

Two-photon excitation (TPE) microscopy[21] uses pulsed mode-locked lasers to excite a femto-litre sized volumes in a specimen via the simultaneous absorption of two photons in the near-infrared range. Two-photon lasers can also image certain collagen types that emit a label-free second harmonic signal (SHG)[22–24]; the resulting signal has a wavelength that is precisely half of the incident laser wavelength. Since near-infrared wavelengths experience less attenuation and contribute to less phototoxicity, TPE is a preferred method for tissue and intravital imaging. Furthermore, multiple fluorophores can share similar two-photon excitation spectra, and this creates a throughput advantage in thick specimens. Recent research has shown that 3-photon excitation (3PE) microscopy enables even deeper imaging than TPE and is also compatible with live cell imaging[25]. However, TPE and 3PE systems are less common than confocal microscopes and require specialist knowledge to operate and maintain. We exploit the optical setup used in TPE in the current manuscript to perform SHG imaging of collagen fibres that are major constituents of the extracellular matrix. We anticipate that other uses of TPE will emerge in tissue profiling but not as a general-purpose means of performing cyclic tissue imaging. Total internal reflection fluorescence (TIRF) microscopy[26,27] can also be used produce 3D images in conjunction with techniques such as DNA paint, but such methods are limited to a depth of ~100 nm from the coverslip.

2. Selecting an optimal 3D imaging approach.

Key considerations in the collection of 3D images from tissue are plex, resolution, and specimen thickness. Optimizing these involves balancing potentially incommensurate variables such as (i) properties of the objective lens with respect to resolution and field of view (ii) contrast and sensitivity (iii) acquisition time and dataset size and (iv) the compatibility of reagents and instruments with cyclic staining and FFPE fixation. With modern instrumentation and cameras, the first three factors depend on predictable physical principles such as the intrinsic efficiency of photon collection and the extent of rejection or restoration of out-of-focus light (e.g. with a pinhole). Compatibility with cyclic methods depends on tissue processing and staining protocols, most of which remain to be developed for FFPE tissue. A final consideration for clinical

specimens is the availability of tissue; as a general rule, histopathology biobanks require that tissue blocks not be exhausted. For human studies, this commonly places an upper limit on the thickness of what can be used (~50-200 microns) for diagnostic specimens.

This paper is primarily focused on imaging methods than overcome the fragmentation of cells observed in conventional cut@5μm tissue sections. We have established that sections ~4-8 fold thicker (30 to 50 μm thick in hydrated form) achieve this while also meeting the requirement of being relatively tissue sparing. For specimens this thick, reconstruction from optical sections collected using confocal microscopy is an ideal 3D imaging approach from a technical perspective (see below) and it is also very widely available on commercial and popular platforms. We have also established that FFPE specimens up to several mm thick can be imaged by LSFM, remounted after imaging, and then sectioned at ~50μm thickness, thereby marrying the high resolution of confocal imaging with the ability of LSFM to coarsely image large tissue structures but at higher throughput.

2.1 Impact of specimen thickness on image quality

Thick tissue specimens (hundreds of microns) can also be imaged by confocal microscopy with longer working distance objectives. **Figure SN1.1** illustrates this for a 400 μm thick section of a human colorectal cancer with adjacent normal tissue following tissue clearing.[10,28] The specimen was immuno-stained for βIII-tubulin (TUBB3), counterstained with DAPI, and then cleared by simple immersion (refractive index = 1.52). Imaging was performed on a Zeiss LSM 980 confocal microscope using a 20×/0.8 Plan-Apochromat air objective (working distance: 0.55 mm), with voxel dimensions of 0.283 μm (XY) and 0.55 μm (Z). Fairly close to the objective lens (at a depth of 16.5 μm), images of nuclei and neuronal processes (the structures primarily stained by anti-TUBB3 antibodies in this specimen) were sharp and well resolved. However, further down the specimen (at a depth of ~216 μm) optical aberrations were evident and the images were dimmer, most likely as a result of scattering of incident and emitted light within the specimen. This and similar images demonstrate that the quality of confocal images falls as specimens get thicker, justifying our selection of 30-50 μm specimen thickness for the bulk of this paper.

3. Visualizing 3D data

It is difficult to represent 3D high-plex image data of tens of thousands of cells using the 2D format of a manuscript figure. The images we show are, therefore, processed in various ways to maximize intelligibility. Quantitative data analysis was performed on primary data (following denoising and background correction) over the entirety of an image unless otherwise specified. The appearance of a 3D object composed of multiple optical planes differs depending on how many planes are shown (one vs a subset vs the full stack) and whether maximum intensity or

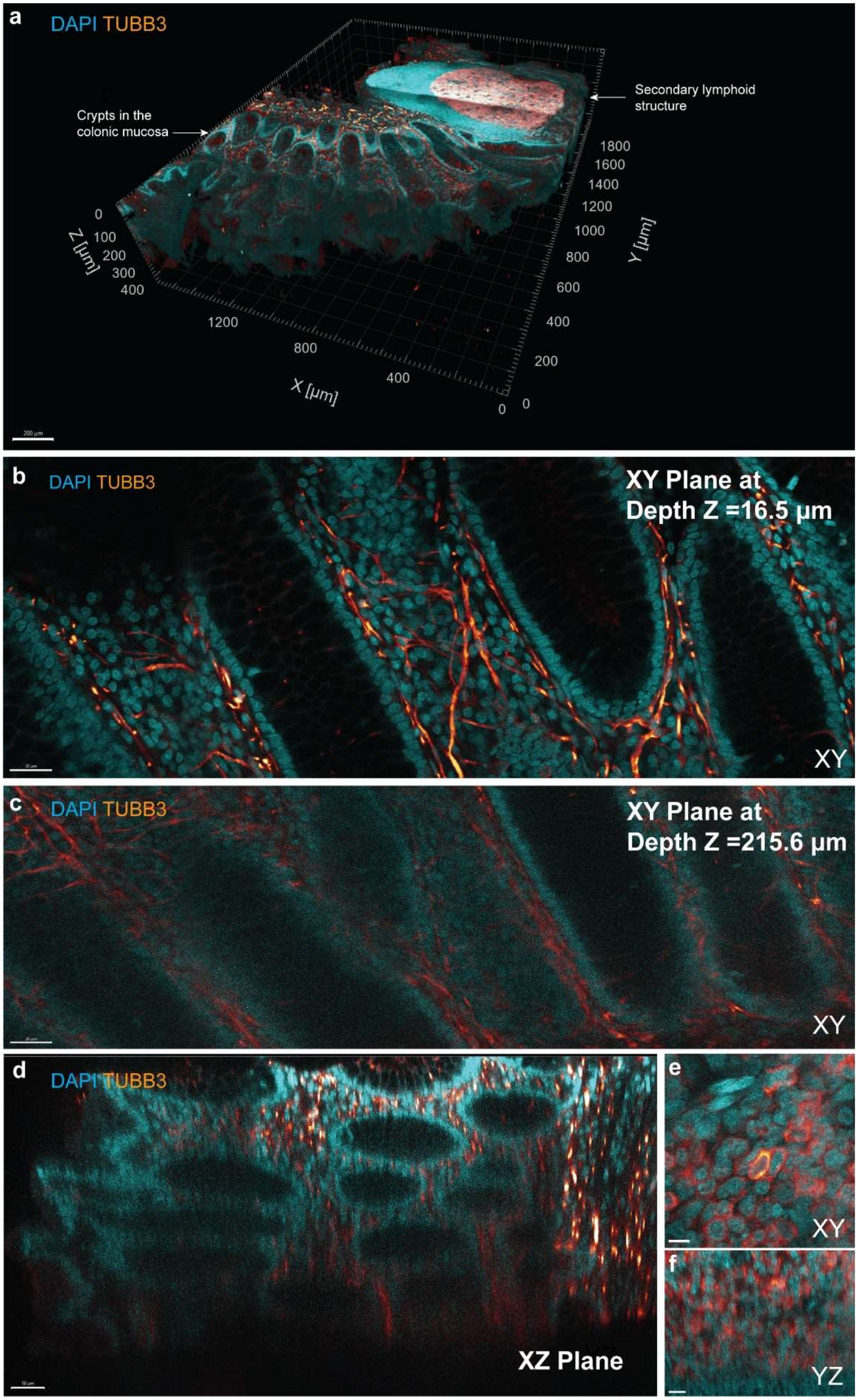

Figure SN1.1: Optical Aberrations in Cleared Tissue Using High-Resolution Confocal Imaging Confocal imaging of optically cleared human colon tissue. **(a)** 3D rendering of a 400 µm-thick cleared colon specimen, showing the organization of neuronal structures stained by βIII-Tubulin (TUBB3; orange) with DNA counter-stained with DAPI (blue). Scale bar: 200 µm. **(b)** Optical section at 16.5 µm depth, showing excellent lateral resolution near the surface. Scale bar: 30 µm. **(c)** Optical section at 215.6 µm depth, with visible degradation in signal and contrast due to refractive index mismatch and light attenuation. Scale bar: 30 µm. **(d)** YZ projection of the full stack, highlighting loss of intensity and resolution beyond ~250 µm along the Z axis. This comes at the cost of increased optical aberrations when imaging deep into high-RI, thick cleared tissue. Scale bar: 50 µm. **(e)** Cropped Image of TUBB3 expressing cells in lymphoid aggregate, Scale bar: 10 µm. **(f) YZ** projection of the lymphoid aggregate with TUBB3 expressing Cells. Scale bar: 50 µm

cell surface renderings are used (**Figure SN1.2**). No approach is objectively superior and in the current paper, each figure panel involves use of a visualization mode intended to illustrate a specific point; this was empirically selected by several people working in parallel based on

viewing the primary 3D data in various projections and rotations. Any questions about these visualizations, which are necessarily incomplete, should be resolved by inspection of the primary data at full resolution.

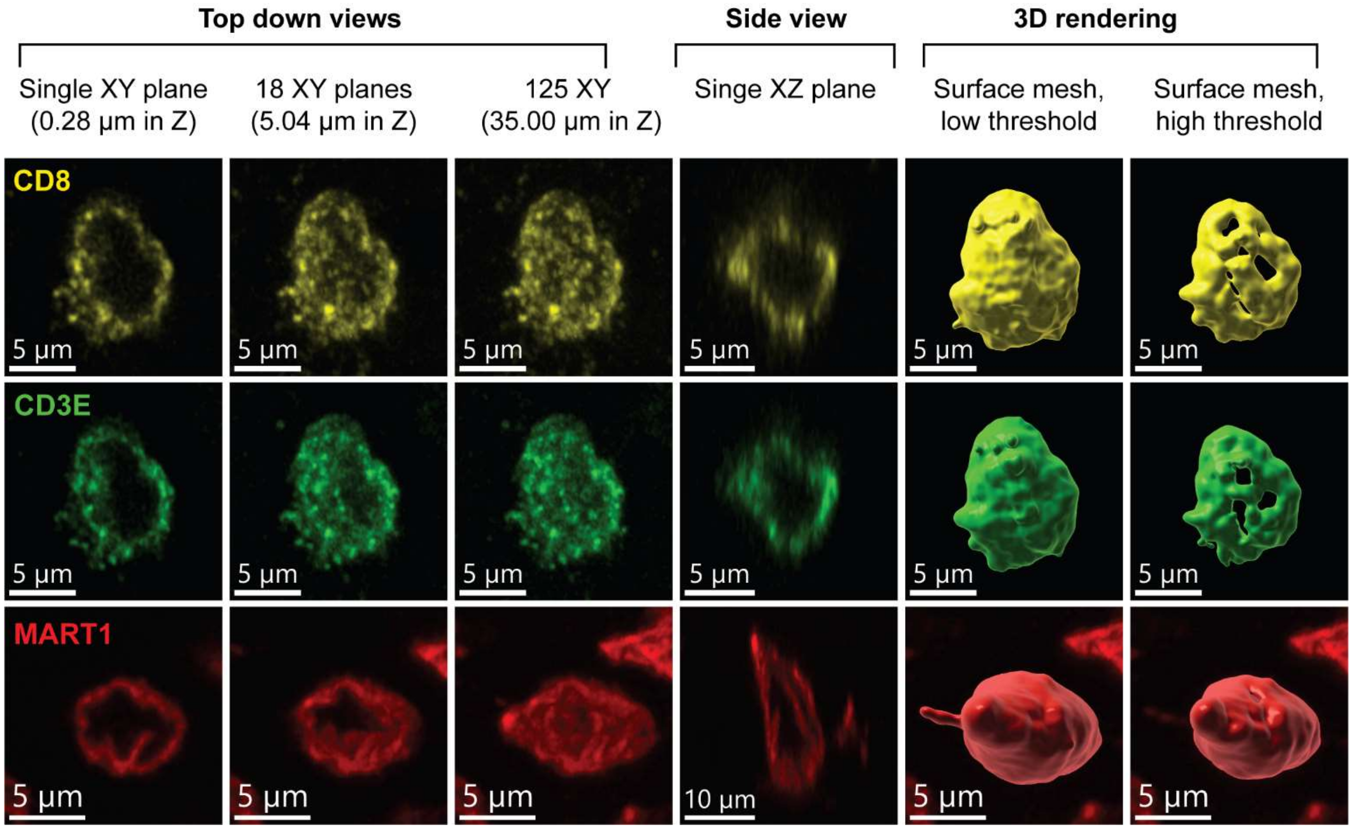

Figure SN1.2: Different ways of representing a cell imaged in 3D. Comparison of different ways of depicting data from a T cell (top two rows) or a tumor cell (bottom row) in a 3D image of a melanoma specimen. A single channel from a 54-plex image of a single cell is shown in each image; other channels and cells have been removed for clarity. The underlying data for all images in each row is the same; only the number of optical sections and the rendering method differ. CD8 is shown in yellow, CD3E in green and the melanocytic marker MART1 in red. Columns represent (left to right): a single optical XY plane-plane representing ~0.28 µm of sample thickness (plus any contribution from out-of-focus light above and below), a maximum intensity projection along the Z axis (optical axes) of 18 or 125 XY planes representing 5.04 µm and 35.00 µm of specimen thickness respectively, a single 0.28 µm thick side view (an XZ plane) demonstrating a reduction in resolution relative to an XY plane. 3D surface rendering in Imaris using either a low threshold (1000 GLU) or a high threshold (2000 GLU). Images were acquired using a 40x/1.3NA oil objective lens on Zeiss LSM980 laser scanning confocal microscope with the voxel size set to 0.14 µm (XY) × 0.28 µm (Z). Scale bar: 5 µm unless indicated.

4. Membrane-Membrane interaction analysis

To estimate the distance between membranes of adjacent cells we used line intensity profiles, which integrate the fluorescence intensity in each channel along a spline parallel to the local axis of the membrane (dimension Y) over a perpendicular distance of 5-10 pixels (0.7 to 1.4 µm along dimension X) and then plotting the resulting value as a function of distance perpendicular to the membrane (dimension X) over a distance of 20-40 pixels (2.8 to 5.6 µm). This approach increased the signal to noise ratio relative to a line integral that was only one pixel wide. The position and distance over which membrane intensity profiles were computed is depicted by

rectangles in **Figure 3** & **6** and **Figure SN1.3-1.5**. Both the position and dimension of the rectangles were determined visually based on the extent of cell-cell contact with the goal of characterizing local contacts at reasonable SNR. We then performed polynomial curve fitting on the resulting profiles to estimate the distance between the peaks of membrane staining. The first and second derivatives were obtained from the fitted curves to determine the roots, and the root at the maximum peak of the fitted curve was used to localise the cell membrane more precisely. As described previously, sub-pixel precision can be obtained using this approach[29]. To determine the robustness of these estimates to limitations in the imaging data we performed a range of studies on prototypical membrane-membrane interactions, which are described below.

4.1 Effect of spatial sampling rate (pixel size) on interaction analysis

We imaged panCK (green) and CD11c (magenta) (**Figure SN1.3a**) at lateral pixel sizes optimal for Nyquist sampling and denoising (60 nm) and then systematically reduced the sampling rate to 600 nm. We found that membrane midpoints could still be identified using polynomial curve fitting. We conclude that our spatial sampling rate (140 nm pixel size for most datasets) is sufficient for estimation of intermembrane distances in this manuscript.

4.2 Effect of magnification and numerical aperture

We compared a high numerical aperture oil immersion lens (40x/1.3NA) to an air objective lens (20x/0.8NA) for the same set of stained cells. Since the sensitivity of an objective lens is dependent on NA and magnification, images collected using a 20x air objective lens were 3-fold dimmer. Despite this, membrane interactions were still detectable by curve fitting of line profiles (**Figure SN1.3b**). We surmise that this arises because out-of-focus light, which significantly impacts precision, is still rejected by the confocal pinhole (which was set to 1 Airy unit in both cases). We conclude that lower magnification and numerical do not significantly impact the estimates of intermembrane distance in this paper.

4.3 Effect of averaging over multiple line profiles

When the signal across a membrane is discontinuous or noisy, averaging several parallel line profiles will smoothen the intensity data. This is more readily implementable across straight membranes than round membranes as in the case of cells from **Figure SN1.4a** (left) imaged with a 40x/1.3NA objective lens with a 1 Airy Unit pinhole for which 10 parallel one-pixel wide line profiles were averaged. However, depending on the severity of the noise, peaks are still identifiable via curve fitting of a single line profile (**Figure SN1.4a-right**).

4.4 Effect of averaging over multiple Z-planes

We compared the use of a single line profile averaged across multiple Z-planes from the pair of cells in **Figure SN1.3b** imaged with a 40x/1.3NA objective lens. Unexpectedly, we found that precision was reduced when multiple Z-planes were used (**Figure SN1.4b**). We surmised that this arose because not all planes fully included the interaction and that including more planes reduced precision. This can be observed by a widening of both membrane intensity curves,

which we observed beyond a thickness of 2.8 microns (10 planes spaced at 280 nm each). Despite this, curve fitting was still able to robustly identify peaks. In general, we attempted to measure intermembrane distance of juxtracrine signalling complexes (Type I interactions) at the point where cells were most closely apposed.

4.5 Effect of poor antibody staining resulting in low signal-to-noise ratio

We sought to assess the effect of using antibodies with low specificity or poor fluorophore selection. This may be observed by higher background and/or lower signal, thus contributing to poorer SNR. **Figure SN1.5a** demonstrates a PD1 and PDL1 interaction on two cells (PD1 conjugated to Alexafluor 647 - magenta; PDL1 conjugated to Alexafluor 647 in a different cycle - yellow). When anti-PD1 antibody was instead conjugated to phycoerythrin (green), a less photostable fluorophore, the overall signal intensity was significantly lower causing the line profile to be noisier. This is evidenced by the multiple peaks along the trace despite performing denoising operations. The peak-to-peak distance increased from 70 nm to 230 nm suggesting that the membranes were incorrectly identified as lying further apart. We conclude that analysis of membrane-membrane interaction requires “good” antibody staining; in the current manuscript we judged this visually, but in future work it should be possible to come develop a more objective metric.

4.6 Effect of out-of-focus rejection

The effect of low signal-to-background due to out-of-focus signal rejection was compared at 20x/0.8NA air and 40x/1.3NA oil immersion lens. For both magnifications, the out-of-focus signal from different optical planes masked the signal from the in-focus plane containing the cell membrane interactions (**Figure SN1.5b**). This was evidenced by the high background signal level. Curve fitting was not able to find the correct peaks with poor signal-to-background ratio. From this, we conclude that the identification of membrane-membrane interactions in thick tissue requires high-resolution 3D data with good rejection of out-of-focus light.

4.7 Summary

In summary, we found that changes in contrast has the most significant impact on assessing cell membrane interactions. Achieving sufficient contrast required rejection of out-of-focus light and strong antibody staining (bright fluorophores, specific antibodies). In the current work we opted to measure membrane intensity profiles along one or a small number of optical planes as this was observed to improve peak height as compared to using entire cell volumes. More specifically, we found that the precision of detecting membrane interactions increases as the thickness of each optical plane decreases but only up to a certain point (beyond that, insufficient signal would be collected). Through the use of curve fitting of membrane intensity profiles, the sampling rate/pixel size could be relatively coarse (e.g. 280 nm in the interest of throughput) for analysis of tightly apposed membranes, but at the cost of less accurate characterization of cell morphology. Overall, we judge that the conditions used in this paper represent a reasonable

starting point for the development of automated proximity detection algorithms based on membrane contact rather than nuclear centroids.

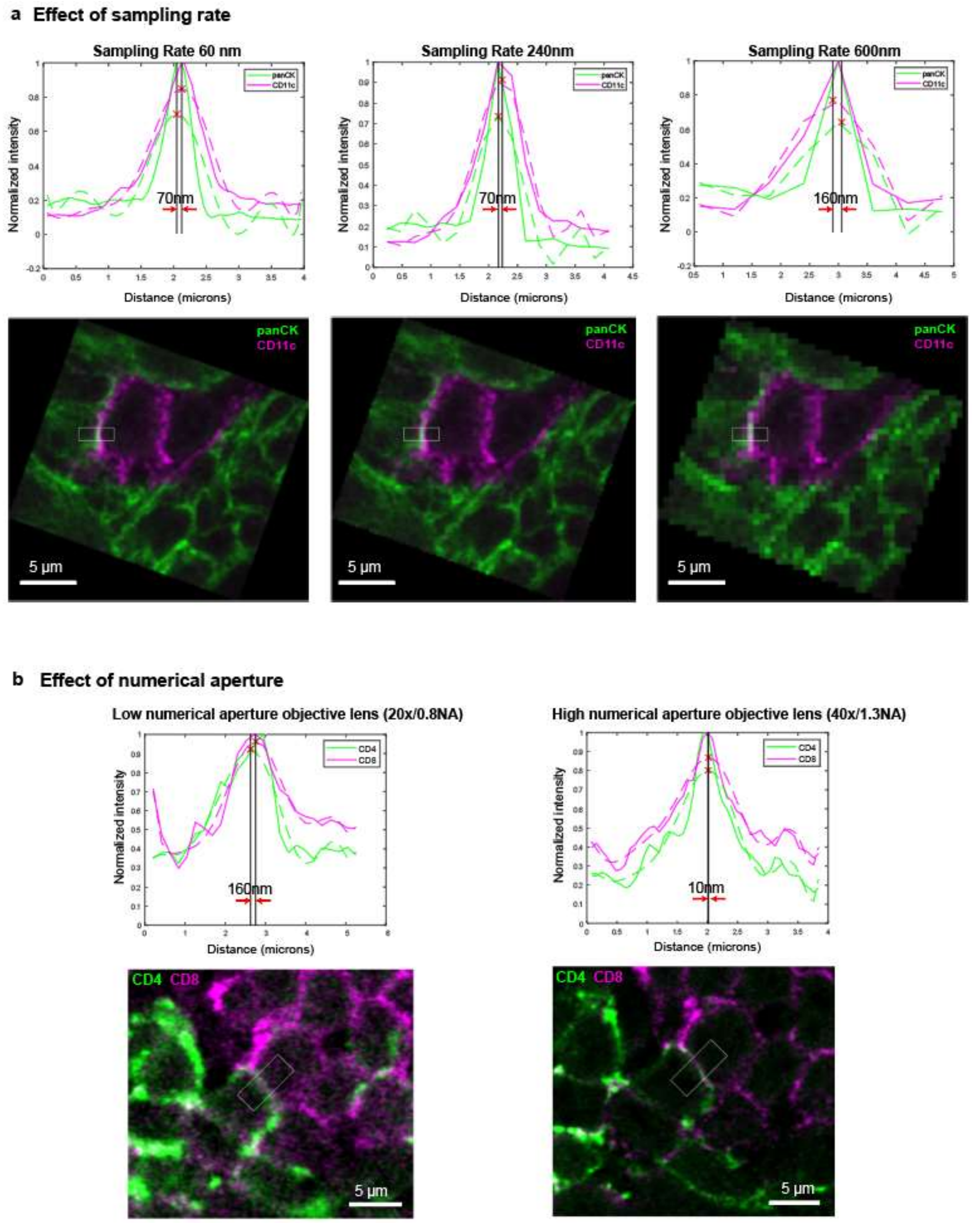

Figure SN1.3: Effect of sampling rate (pixel size) and optical resolution (NA) on image quality and ability to identify cell-to-cell membrane contacts. a, dramatic reduction in sampling rate (increasing pixel size from 60nm to 600nm) had a surprisingly small marginal impact on the ability to detect membrane peaks from line profiles. panCK – green, CD11c – magenta. Box indicates region that line profiles were extracted from. Tissue prepared and stained on glass slide at 4 degrees C overnight, washed in PBS and mounted with 70% glycerol. Imaged with 40x/1.3NA oil objective lens on Zeiss LSM980 laser scanning confocal microscope sampled at 140nm (x,y). Scalebar 5 µm. **b,** Line profiles and single optical z-planes of two interacting immune cells in a 5-micron thick colorectal cancer sample imaged at 20x/0.8NA (left) and 40x/1.3NA (right) objective lens. Using a lower numerical aperture objective (20x/0.8NA) results in lower photon sensitivity and a noisier image. Peak detection from line profiles is still possible. CD4 – green, CD8 – magenta.

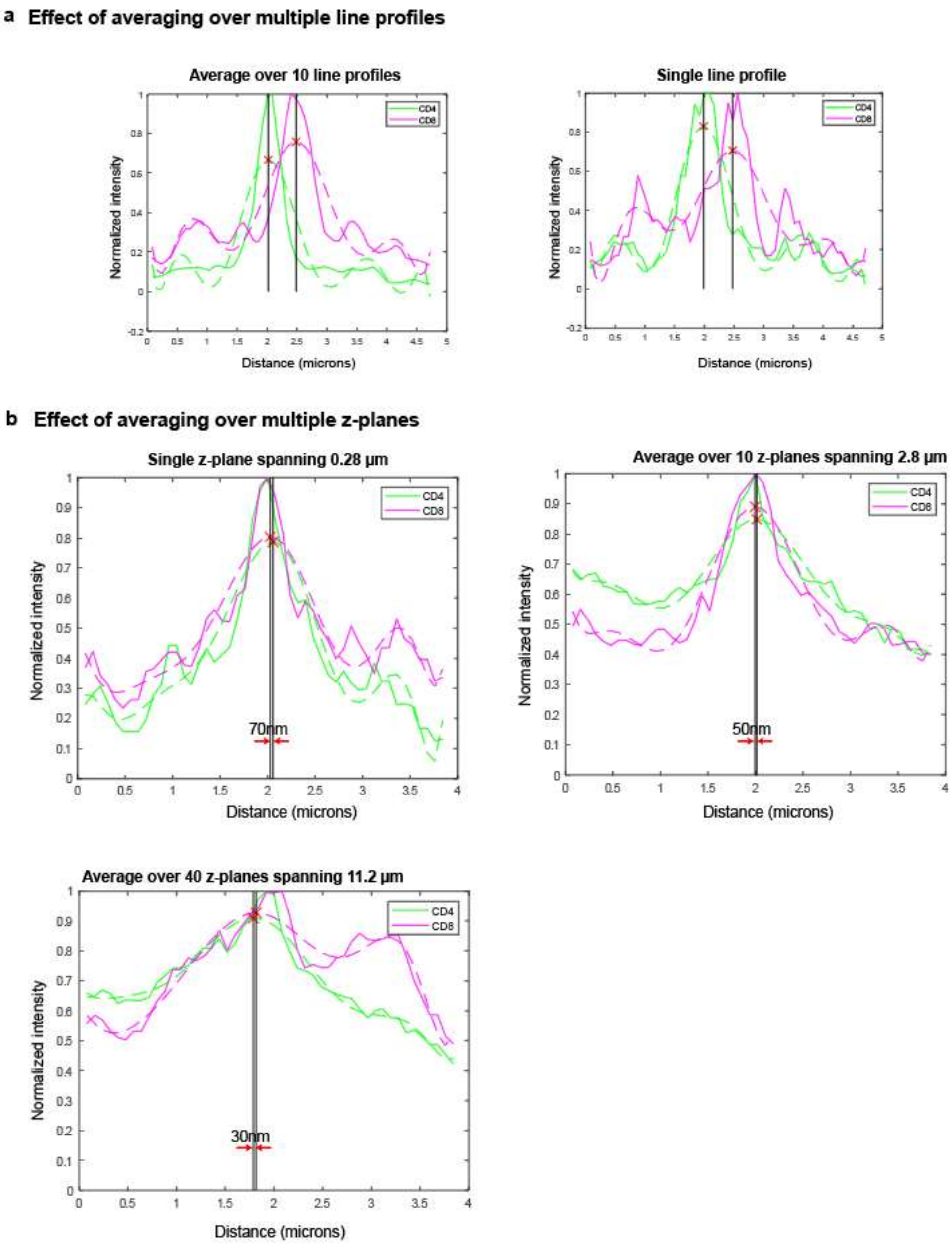

Figure SN1.4: Effect of different line profile measurement methods on ability to identify cell to cell membrane contacts. a, Comparison of line profiles taken from 10 parallel lines (left) vs 1 line (right) across the membrane of two neighbouring cells from **Figure SN1.3b**. A single line profile is noisier than averaging multiple line profiles but accurate peak detection is still possible. **b,** Single line profiles across 1, 10, and 40 z-planes in two cells (**Figure SN1.3b)** are compared. Thicker z-planes may extend beyond the actual site of interaction thereby introduce noise and signal from other cells. CD4 – green, CD8 – magenta. Tissue prepared and stained on glass slide at 4 degrees C overnight, washed in PBS and mounted with 70% glycerol. Imaged on a Zeiss LSM980 laser scanning confocal microscope sampled at 140nm (x,y) and 280nm (z).

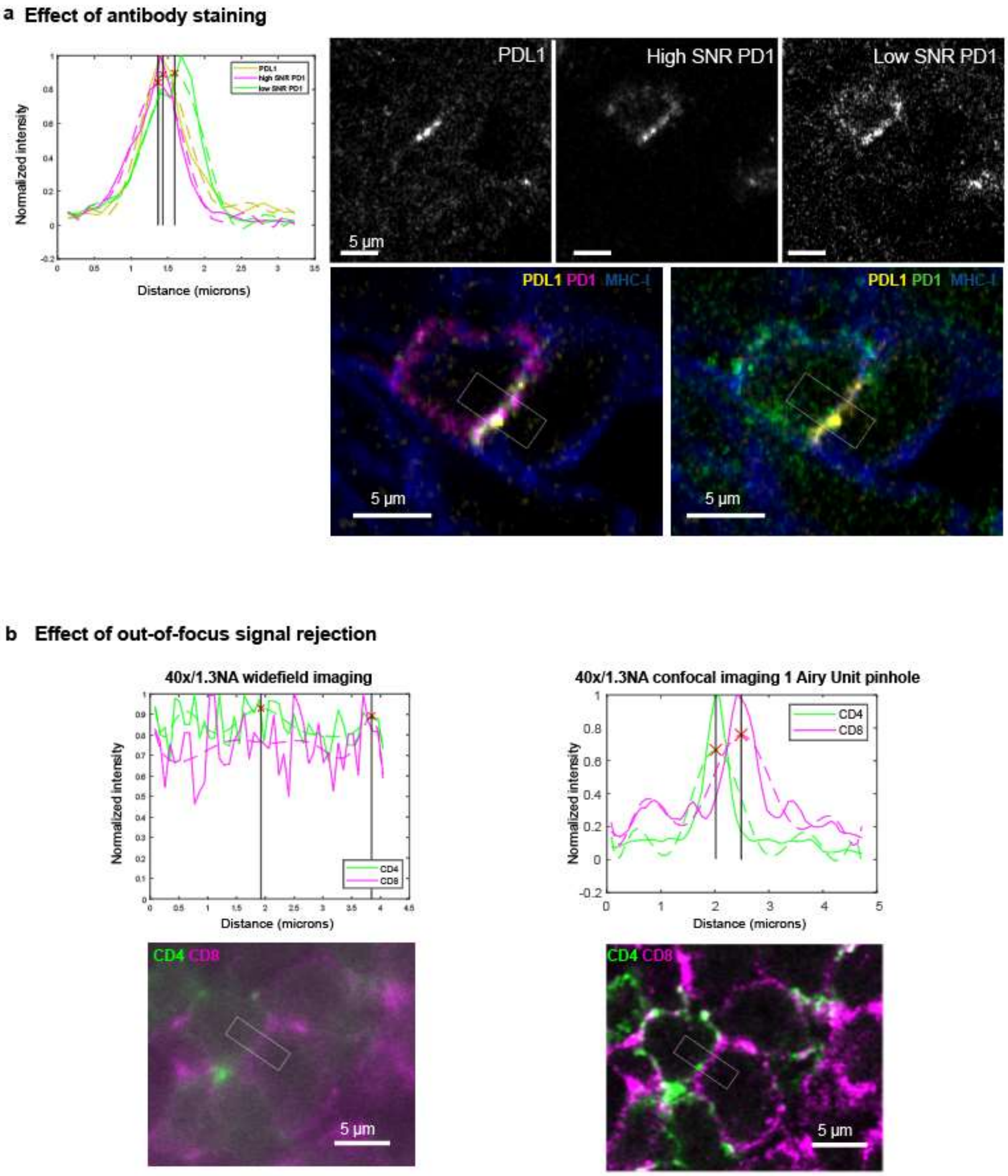

Figure SN1.5: Effect of confocality and antibody signal on ability to identify membrane- membrane contacts. a, Line profiles and single optical sections (z-planes) of two interacting cells stained with PDL1 (yellow), high SNR PD1 (magenta) and low SNR PD1 (green) in a 5-micron thick colorectal cancer tissue. MHC-1 shown in blue to demarcate cell membranes. The peak for low SNR PD1 has shifted due to noise. **b,** Line profiles and fluorescence images comparing widefield microscopy (left) and confocal imaging at 1 Airy Unit pinhole (right) on the exact same cells. Determining peak position in widefield images is hindered due to severe out-of-focus signal. CD4 – green, CD8 – magenta. Box indicates region that line profiles were extracted from. Tissue prepared and stained on glass slide at 4 degrees C overnight, washed in PBS and mounted with 70% glycerol. Imaged on a Zeiss LSM980 laser scanning confocal microscope sampled at 140nm (x,y). Scalebar 5 µm.

5. Discriminating punctate from diffuse signals in thick tissues using confocal microscopy

In the previous sections of this Supplementary Note, we discussed 3D imaging methods and discussed why it is important to reject out-of-focus blur (a feature of confocal microscopy) and

optimize sampling rate, objective lens, and antibody selection. In this section, we consider the resolution of a membrane-bound juxtracrine signalling complex involving PD1 and PDL1. Interaction of these proteins supresses T cell activity and is the target of most immunotherapy, making it an interaction of high interest. Both proteins had very variable staining morphologies, appearing as both bright puncta or diffuse signals. This contrasted with proteins such as MART1 that were diffuse through the cell membrane. The question arose in review whether our representations of PD1 and PDL1 staining were accurate of the underlying data and whether imaging artefacts contribute to differences in observed morphology.

We therefore collected images of PD1 and PDL1 in immune and tumour cells in a variety of contexts including vertical growth phase melanoma, (**Figure SN1.6**), melanoma in situ (**Figure SN1.7**)**,** and metastatic melanoma (**Figure SN1.8 & 1.9**) and then processed the images in multiple ways**.** With the exception of the melanoma in situ sample, we observed PDL1 staining in a large population of dendritic cells as shown by the 2nd column in **Figure SN 1.6, 1.8 and 1.9**. PDL1 puncta also varied from uniform distribution within a cell to polarized towards neighbouring cells; for example, at points of contact between a CD8 T cell and proximate tumour cell (**Figure SN 1.7 –** also shown in **Figure 6a).** In these cases, we found that the intensities of both punctate and diffuse PD1 and PDL1 were well above the background arising from detector noise and autofluorescence (>1000 GLUs above background). Moreover, puncta were easily detectable using classical spot detection methods or simple line profiles. As shown in Figure **SN 1.6B-i** and Figure **SN 1.8B-i**, line profiles can readily resolve PDL1 and PD1 and confirm their punctate morphology.

We conclude that our data do show both punctate and diffuse staining for PD1 and PDL1 and that our figures are reasonable representations of the underlying data.

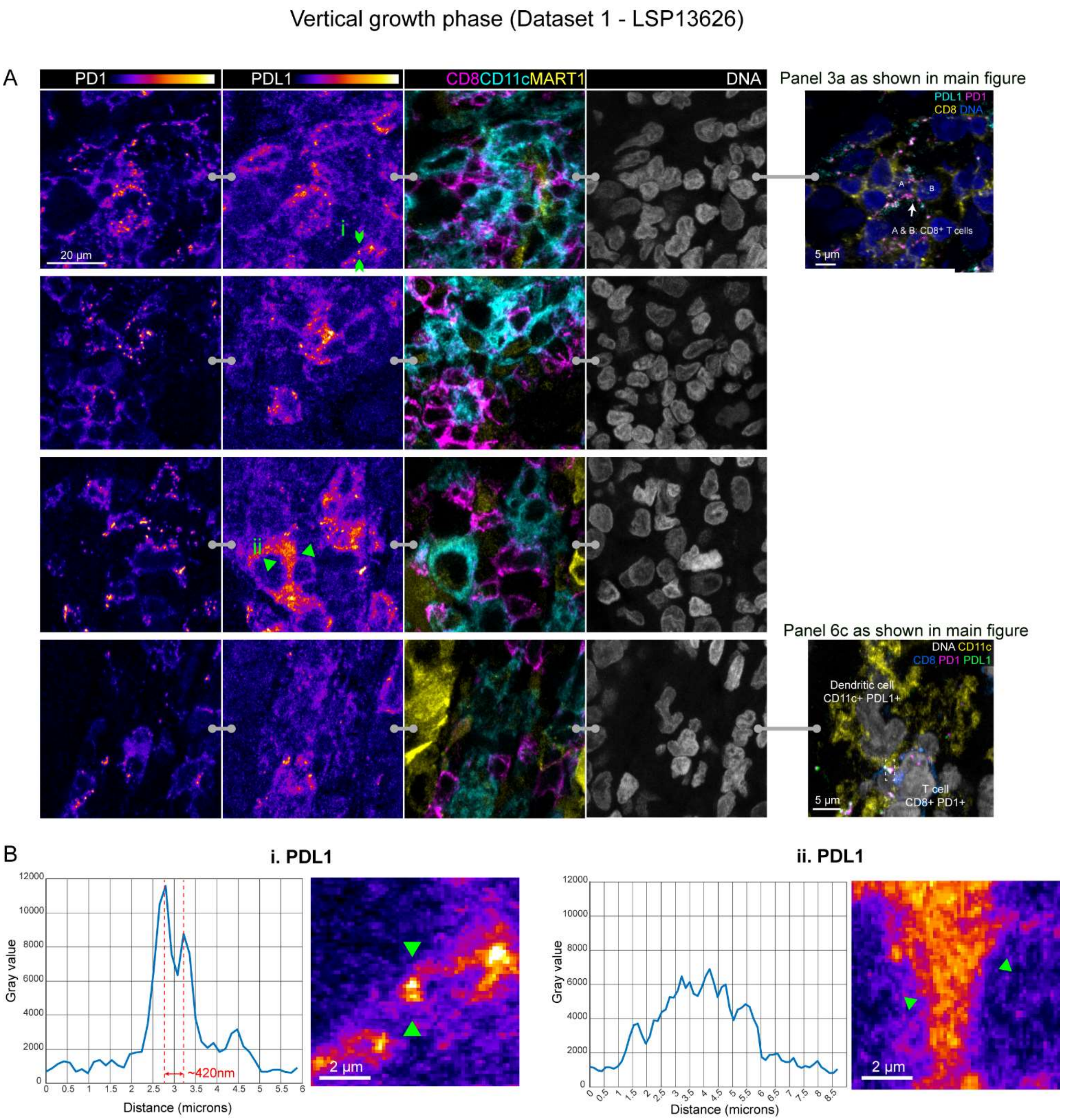

Figure SN 1.6: 5-micron maximum intensity projections comparing punctate and diffuse morphology of PD1 and PDL1 in the vertical growth phase. PD1 and PDL1 shown with 'Fire' LUT. Green arrowheads indicate extent of corresponding line intensity profiles depicted in A. Combined channels of CD8, CD11c, and MART1 also shown in magenta, cyan, and yellow respectively and DNA (grey). Scale bar is 20 microns. Panels of corresponding main figures are indicated. B) Line intensity profiles over select PDL1 cells indicated by green arrowheads in A. Inset shows zoomed in view of region indicated by green arrowheads. Scale bar is 2 microns. B-i) Signal intensity along line profile shows distinct high-intensity peaks (5,000-10,000 GLUs above background) indicating PDL1 concentrating into puncta. B-ii) Line intensity profiles across diffuse PDL1 shows wider profile with noise amplitude of approximately less than 5,000 GLU above background.

Melanoma in-situ (Dataset 1 - LSP13626)

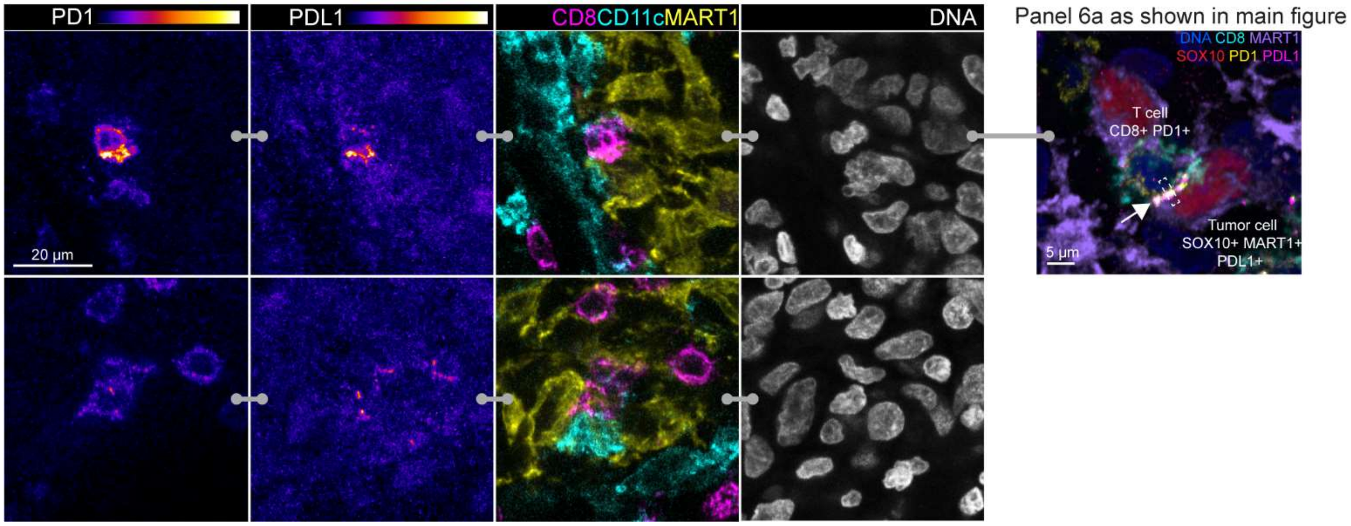

Figure SN 1.7: 5-micron maximum intensity projections comparing punctate and diffuse morphology of PD1 and PDL1 in melanoma in situ. PD1 and PDL1 shown with ‘Fire’ LUT. Combined channels of CD8, CD11c, and MART1 also shown in magenta, cyan, and yellow respectively and DNA (grey). Scale bar is 20 microns. Panels of corresponding main figures are indicated.

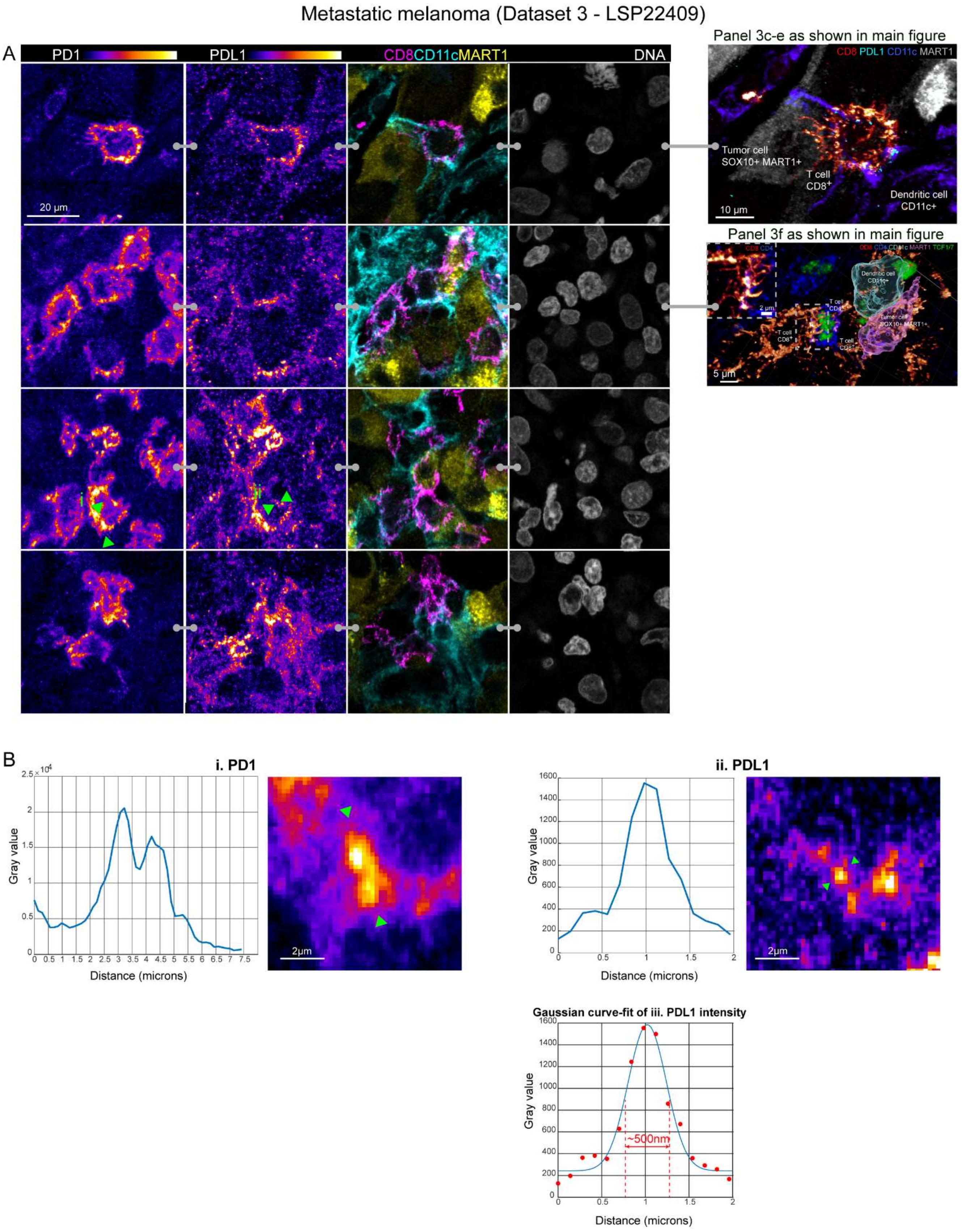

Figure SN 1.8: 5-micron maximum intensity projections comparing punctate and diffuse morphology of PD1 and PDL1 in metastatic melanoma. PD1 and PDL1 shown with 'Fire' LUT. Green arrowheads indicate extent of corresponding line intensity profiles depicted in A. Combined channels of CD8, CD11c, and MART1 also shown in magenta, cyan, and yellow respectively and DNA (grey). Scale bar is 20 microns. Panels of corresponding main figures are indicated. B) Line intensity profiles over select PD1 and PDL1 cells indicated by green arrowheads in A. Inset shows zoomed in view of region indicated by

green arrowheads. Scale bar is 2 microns. B-i) Signal intensity along line profile shows distinct high-intensity peaks (10,000-15,000 GLUs above background) indicating PD1 concentrating into puncta. B-ii (top) Line intensity profiles across single PDL1 punctum with distinct high-intensity peak (1000 GLUs above background). B-ii (bottom) Gaussian-fitted curve (blue) overlaid over raw PDL1 signal (B-i-top) shown as red dots. Full-width-half-maximum of signal indicates width of PDL1 punctum is approximately 500nm.

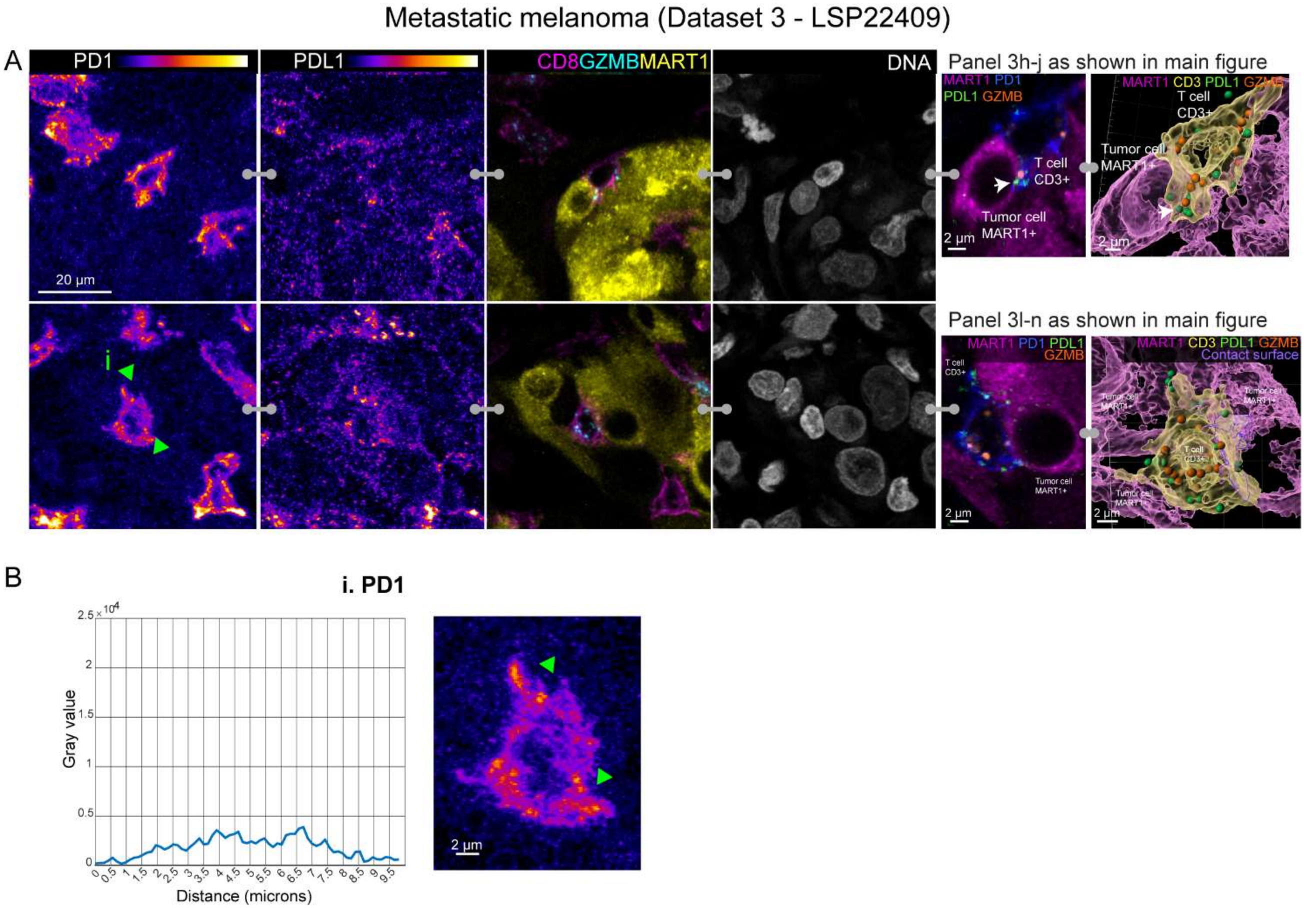

Figure SN 1.9: 5-micron maximum intensity projections comparing punctate and diffuse morphology of PD1 and PDL1 in metastatic melanoma. PD1 and PDL1 shown with 'Fire' LUT. Green arrowheads indicate extent of corresponding line intensity profiles depicted in A. Combined channels of CD8, GZMB, and MART1 also shown in magenta, cyan, and yellow respectively and DNA (grey). Scale bar is 20 microns. Panels of corresponding main figures are indicated. B) Line intensity profiles over select PD1 cells indicated by green arrowheads in A. Inset shows zoomed in view of region indicated by green arrowheads. Scale bar is 2 microns. B-i) Signal intensity along line profile shows wider profile with noise amplitude of approximately less than 5,000 GLU above background.

6. References

1. Kuett, L. *et al.* Three-dimensional imaging mass cytometry for highly multiplexed molecular and cellular mapping of tissues and the tumor microenvironment. *Nat. Cancer* **3**, 122–133 (2022).

2. Ghose, S. *et al.* 3D reconstruction of skin and spatial mapping of immune cell density, vascular distance and effects of sun exposure and aging. *Commun. Biol.* **6**, 718 (2023).

3. Lin, J.-R. *et al.* Multiplexed 3D atlas of state transitions and immune interaction in colorectal cancer. *Cell* **186**, 363-381.e19 (2023).

4. Wallace, W., Schaefer, L. H. & Swedlow, J. R. A Workingperson's Guide to Deconvolution in Light Microscopy. *BioTechniques* **31**, 1076–1097 (2001).

5. Nirmal, A. J. *et al.* The Spatial Landscape of Progression and Immunoediting in Primary Melanoma at Single-Cell Resolution. *Cancer Discov.* **12**, 1518–1541 (2022).

6. Egger, M. D. & Petráň, M. New Reflected-Light Microscope for Viewing Unstained Brain and Ganglion Cells. *Science* **157**, 305–307 (1967).

7. Wilson, T. Resolution and optical sectioning in the confocal microscope: *J. Microsc.* **244**, 113–121 (2011).

8. Wilson, T. Optical sectioning in confocal fluorescent microscopes. *J. Microsc.* **154**, 143–156 (1989).

9. Murray, J. M., Appleton, P. L., Swedlow, J. R. & Waters, J. C. Evaluating performance in three-dimensional fluorescence microscopy. *J. Microsc.* **228**, 390–405 (2007).

10. Li, W., Germain, R. N. & Gerner, M. Y. Multiplex, quantitative cellular analysis in large tissue volumes with clearing-enhanced 3D microscopy (Ce3D). *Proc. Natl. Acad. Sci. U. S. A.* **114**, E7321–E7330 (2017).

11. Renier, N. *et al.* iDISCO: A Simple, Rapid Method to Immunolabel Large Tissue Samples for Volume Imaging. *Cell* **159**, 896–910 (2014).

12. Tomer, R., Ye, L., Hsueh, B. & Deisseroth, K. Advanced CLARITY for rapid and high-resolution imaging of intact tissues. *Nat. Protoc.* **9**, 1682–1697 (2014).

13. Tanaka, N. *et al.* Whole-tissue biopsy phenotyping of three-dimensional tumours reveals patterns of cancer heterogeneity. *Nat. Biomed. Eng.* **1**, 796–806 (2017).

14. Li, W., Germain, R. N. & Gerner, M. Y. High-dimensional cell-level analysis of tissues with Ce3D multiplex volume imaging. *Nat. Protoc.* **14**, 1708–1733 (2019).

15. Murray, E. *et al.* Simple, Scalable Proteomic Imaging for High-Dimensional Profiling of Intact Systems. *Cell* **163**, 1500–1514 (2015).

16. Chen, B.-C. *et al.* Lattice light-sheet microscopy: Imaging molecules to embryos at high spatiotemporal resolution. *Science* **346**, 1257998 (2014).

17. Dean, K. M., Roudot, P., Welf, E. S., Danuser, G. & Fiolka, R. Deconvolution-free Subcellular Imaging with Axially Swept Light Sheet Microscopy. *Biophys. J.* **108**, 2807–2815 (2015).

18. Ku, T. *et al.* Multiplexed and scalable super-resolution imaging of three-dimensional protein localization in size-adjustable tissues. *Nat. Biotechnol.* **34**, 973–981 (2016).

19. Park, J. *et al.* Epitope-preserving magnified analysis of proteome (eMAP). *Sci. Adv.* **7**, eabf6589 (2021).

20. Saka, S. K. *et al.* Immuno-SABER enables highly multiplexed and amplified protein imaging in tissues. *Nat. Biotechnol.* **37**, 1080–1090 (2019).

21. Denk, W., Strickler, J. H. & Webb, W. W. Two-Photon Laser Scanning Fluorescence Microscopy. *Science* **248**, 73–76 (1990).

22. Campagnola, P. J. *et al.* Three-Dimensional High-Resolution Second-Harmonic Generation Imaging of Endogenous Structural Proteins in Biological Tissues. *Biophys. J.* **82**, 493–508 (2002).

23. Campagnola, P. J., Wei, M., Lewis, A. & Loew, L. M. High-Resolution Nonlinear Optical Imaging of Live Cells by Second Harmonic Generation. *Biophys. J.* **77**, 3341–3349 (1999).

24. Zipfel, W. R. *et al.* Live tissue intrinsic emission microscopy using multiphoton-excited native fluorescence and second harmonic generation. *Proc. Natl. Acad. Sci.* **100**, 7075–7080 (2003).

25. Bakker, G.-J. *et al.* Intravital deep-tumor single-beam 3-photon, 4-photon, and harmonic microscopy. *eLife* **11**, e63776 (2022).

26. Fish, K. N. Total Internal Reflection Fluorescence (TIRF) Microscopy. *Curr. Protoc. Cytom.* **50**, (2009).

27. Axelrod, D., Thompson, N. L. & Burghardt, T. P. Total internal reflection fluorescent microscopy. *J. Microsc.* **129**, 19–28 (1983).

28. Chen, F., Tillberg, P. W. & Boyden, E. S. Optical imaging. Expansion microscopy. *Science* **347**, 543–548 (2015).

29. Niederhuber, M. J., Lambert, T. J., Yapp, C., Silver, P. A. & Polka, J. K. Superresolution microscopy of the β-carboxysome reveals a homogeneous matrix. *Mol. Biol. Cell* **28**, 2734–2745 (2017).
