# Supplementary material for: Highly multiplexed 3D profiling of cell states and immune niches in human tumors
Source: Nat Methods. 2025 Sep 29;22(10):2180–93. doi: 10.1038/s41592-025-02824-x (PMC12510885; doi:10.1038/s41592-025-02824-x)
Supplement: Supplementary file 2 — Reporting Summary [file 41592_2025_2824_MOESM2_ESM.pdf]

nature portfolio

Corresponding author(s): Peter Sorger

Last updated by author(s): 04-02-2025

Reporting Summary

Nature Portfolio wishes to improve the reproducibility of the work that we publish. This form provides structure for consistency and transparency in reporting. For further information on Nature Portfolio policies, see our Editorial Policies and the Editorial Policy Checklist.

Statistics

For all statistical analyses, confirm that the following items are present in the figure legend, table legend, main text, or Methods section.

n/a	Confirmed	
☐	☒	The exact sample size (*n*) for each experimental group/condition, given as a discrete number and unit of measurement
☐	☒	A statement on whether measurements were taken from distinct samples or whether the same sample was measured repeatedly
☐	☒	The statistical test(s) used AND whether they are one- or two-sided<br>*Only common tests should be described solely by name; describe more complex techniques in the Methods section.*
☒	☐	A description of all covariates tested
☒	☐	A description of any assumptions or corrections, such as tests of normality and adjustment for multiple comparisons
☐	☒	A full description of the statistical parameters including central tendency (e.g. means) or other basic estimates (e.g. regression coefficient) AND variation (e.g. standard deviation) or associated estimates of uncertainty (e.g. confidence intervals)
☒	☐	For null hypothesis testing, the test statistic (e.g. *F*, *t*, *r*) with confidence intervals, effect sizes, degrees of freedom and *P* value noted<br>*Give P values as exact values whenever suitable.*
☒	☐	For Bayesian analysis, information on the choice of priors and Markov chain Monte Carlo settings
☒	☐	For hierarchical and complex designs, identification of the appropriate level for tests and full reporting of outcomes
☒	☐	Estimates of effect sizes (e.g. Cohen's *d*, Pearson's *r*), indicating how they were calculated

Our web collection on statistics for biologists contains articles on many of the points above.

Software and code

Policy information about availability of computer code

Data collection	Images acquired with Zeiss ZEN 3.7 with LSM Plus Processing.
Data analysis	Image registration and data analysis was performed in Mathworks MATLAB 2021b with Image Processing Toolbox, Curve Fitting Toolbox, Statistics and Machine Learning toolbox, and parallel computing toolbox, Python v3.9 and SCIMAP 1.3.1 (scimap.xyz; https://github.com/labsyspharm/scimap). Cell segmentation performed in Cellpose (https://github.com/MouseLand/cellpose). 3D visualization done in Bitplane Imaris 10.0.

For manuscripts utilizing custom algorithms or software that are central to the research but not yet described in published literature, software must be made available to editors and reviewers. We strongly encourage code deposition in a community repository (e.g. GitHub). See the Nature Portfolio guidelines for submitting code & software for further information.

Data

Policy information about availability of data

All manuscripts must include a data availability statement. This statement should provide the following information, where applicable:

- Accession codes, unique identifiers, or web links for publicly available datasets
- A description of any restrictions on data availability
- For clinical datasets or third party data, please ensure that the statement adheres to our policy

Data Availability (At time of publication)

Code and working demo can be found at https://github.com/labsyspharm/mel-3d-mis. All primary images and derived data (~5 TB) will available via AWS transfer at the time of publication. Instructions for accessing the primary and derived data is available via a data index page on Zenodo (doi.org/10.5281/zenodo.10055593). These images can be viewed using the free Imaris viewer (https://imaris.oxinst.com/imaris-viewer). 2D maximum projections of each dataset can be viewed in the MINERVA viewer (no download required), please see Supplementary Table 1 for links. A subset of data will be available for 3D interactive viewing within the browser-based tool Vitessce (http://vitessce.io/). This effort is a work in progress and will be available in the future.

Research involving human participants, their data, or biological material

Policy information about studies with human participants or human data. See also policy information about sex, gender (identity/presentation), and sexual orientation and race, ethnicity and racism.

Reporting on sex and gender	This study is not human subjects research. Supplementary Table 1 lists patient demographics for the 3 melanoma patients.
Reporting on race, ethnicity, or other socially relevant groupings	Listed in Supplementary Table 1.
Population characteristics	N/A
Recruitment	N/A
Ethics oversight	Specimens were retrieved from the archives of the Department of Pathology at Brigham and Women's Hospital and collected under Institutional Review Board approval (FWA00007071, Protocol IRB18-1363) under a waiver of consent.

Note that full information on the approval of the study protocol must also be provided in the manuscript.

Field-specific reporting

Please select the one below that is the best fit for your research. If you are not sure, read the appropriate sections before making your selection.

☒ Life sciences ☐ Behavioural & social sciences ☐ Ecological, evolutionary & environmental sciences

For a reference copy of the document with all sections, see nature.com/documents/nr-reporting-summary-flat.pdf

Life sciences study design

All studies must disclose on these points even when the disclosure is negative.

Sample size	Dataset 1 and 2 are serial adjacent sections from the same patient. All measurements were taken from regions of interests from unique patient-derived samples, which limits large sample size. Regions encompass the majority of the lesion as identified by board-certified pathologist.
Data exclusions	Images of antibody stains that did not stain/wash/register properly were excluded from analysis but are still included in imaris files for viewing and labeled as such.
Replication	Dataset 1 and 2 are serial adjacent sections from the same patient. Measurements were taken from unique patient-derived samples, and therefore, the possibility of running replication studies is very limited.
Randomization	N/A
Blinding	Conventional blinding is not relevant to this type of retrospective non-interventional study. Details of the model development are described in detail in the text and methods.

Reporting for specific materials, systems and methods

We require information from authors about some types of materials, experimental systems and methods used in many studies. Here, indicate whether each material, system or method listed is relevant to your study. If you are not sure if a list item applies to your research, read the appropriate section before selecting a response.

Materials & experimental systems

n/a	Involved in the study
☐	☒ Antibodies
☒	☐ Eukaryotic cell lines
☒	☐ Palaeontology and archaeology
☒	☐ Animals and other organisms
☒	☐ Clinical data
☒	☐ Dual use research of concern
☒	☐ Plants

Methods

n/a	Involved in the study
☒	☐ ChIP-seq
☒	☐ Flow cytometry
☒	☐ MRI-based neuroimaging

Antibodies

Antibodies used

Clone information and RRID of primary and secondary antibodies used in the manuscript can be found in Supplementary Table 3-10.

1 , Target: Alpha-actin-2 , Label: Alexa Fluor 750 , R&D Systems , Cat: IC1420S-025 , Clone: 1A4 , RRID: AB_2868436
1 , Target: CD11b , Label: Alexa Fluor 488 , Thermo Fisher Scientific (eBioscience) , Cat: 53-0196-80, 53-0196-82 , Clone: C67F154 , RRID: AB_2637195
1 , Target: CD3E , Label: Alexa Fluor 488 , Cell Signaling Technology , Cat: 86936BC , Clone: D7A6E , RRID:
1 , Target: CD45 , Label: Alexa Fluor 647 , BioLegend , Cat: 304020, 304056 , Clone: HI30 , RRID: AB_493034
1 , Target: CD66b , Label: phycoerythrin , BioLegend , Cat: 392903 , Clone: 6/40c , RRID: AB_2750201
1 , Target: COX4 , Label: Alexa Fluor 555 , Abcam , Cat: ab210675 , Clone: EPR9442(ABC) , RRID: AB_2857975
1 , Target: CPT1A , Label: Alexa Fluor 488 , Abcam , Cat: ab171449 , Clone: 8F6AE9 , RRID: AB_2714024
1 , Target: Cytokeratin (pan) , Label: Alexa Fluor 750 , Novus Biologicals , Cat: NBP2-33200AF750 , Clone: AE-1/AE-3 , RRID: AB_2868569
1 , Target: ECP , Label: , Abcam , Cat: ab207429 , Clone: EPR20357 , RRID: AB_2943114
1 , Target: Gamma Tubulin , Label: Alexa Fluor 647 , Abcam , Cat: ab191114 , Clone: TU-30 , RRID: AB_2889219
1 , Target: Goat IgG , Label: Alexa Fluor 555 , Thermo Fisher Scientific , Cat: A-21432 , Clone: , RRID: AB_2535853
1 , Target: Langerin , Label: , R&D Systems , Cat: AF2088-SP , Clone: , RRID: AB_355143
1 , Target: Mouse IgG , Label: Alexa Fluor 647 , Thermo Fisher Scientific , Cat: A-21237 , Clone: , RRID: AB_2535806
1 , Target: MPO , Label: Alexa Fluor 647 , Santa Cruz Biotechnology , Cat: sc-365436-AF647 , Clone: A-5 , RRID: AB_2943296
1 , Target: PCNA , Label: Alexa Fluor 750 , Cell Signaling Technology , Cat: 24114BC , Clone: PC10 , RRID:
1 , Target: PD-L1 , Label: Alexa Fluor 647 , Cell Signaling Technology , Cat: 62813BC , Clone: E1L3N , RRID:
1 , Target: phospho-Histone 3 , Label: Alexa Fluor 750 , Cell Signaling Technology , Cat: 43185BC , Clone: D2C8 , RRID:
1 , Target: Rabbit IgG , Label: Alexa Fluor 488 , Invitrogen , Cat: A-11070 , Clone: , RRID: AB_2534114
1 , Target: S100A1 , Label: , Abcam , Cat: ab183979 , Clone: EPR19013 , RRID: AB_2894716
1 , Target: S6 (Ser235/236) , Label: Alexa Fluor 750 , Cell Signaling Technology , Cat: 62788BC , Clone: D57.2.2E , RRID:
1 , Target: SOX10 , Label: , Abcam , Cat: ab216020 , Clone: SOX10/1074 , RRID: AB_2847913
1 , Target: Stat1 (pY701) , Label: Alexa Fluor 555 , Cell Signaling Technology , Cat: 8183S , Clone: 58D6 , RRID: AB_10860600
1 , Target: TCF1/TCF7 , Label: Alexa Fluor 488 , Cell Signaling Technology , Cat: 6444 , Clone: C63D9 , RRID: AB_2797627
1 , Target: Vinculin , Label: eFluor 570 , Thermo Fisher Scientific , Cat: 41-9777-80 , Clone: 7F9 , RRID: AB_2573646
2 , Target: 5'-HMC , Label: , Active Motif , Cat: 39769 , Clone: , RRID: AB_10013602
2 , Target: Actin, cytoplasmic 1 , Label: Alexa Fluor 555 , Cell Signaling Technology , Cat: 8046S , Clone: 13E5 , RRID: AB_11179208
2 , Target: BANF1 , Label: Alexa Fluor 568 , Abcam , Cat: ab208534 , Clone: EPR7668 , RRID: AB_2868492
2 , Target: Catalase , Label: Alexa Fluor 488 , Abcam , Cat: ab185041 , Clone: EP1929Y , RRID: AB_2884892
2 , Target: CD103 , Label: Alexa Fluor 647 , BioLegend , Cat: 350209 , Clone: Ber-ACT8 , RRID: AB_10640870
2 , Target: CD15 , Label: Alexa Fluor 488 , BioLegend , Cat: 301910 , Clone: HI98 , RRID: AB_493257
2 , Target: CD8a , Label: eFluor 660 , eBioscience , Cat: 50-0008-82 , Clone: AMC908 , RRID: AB_2574149
2 , Target: COX4-1 , Label: Alexa Fluor 647 , Cell Signaling Technology , Cat: 7561S , Clone: 3E11 , RRID: AB_10994876
2 , Target: Cyclin-D1 , Label: Alexa Fluor 488 , Abcam , Cat: AB190194 , Clone: EPR2241 , RRID: AB_2728784
2 , Target: Cytokeratin (pan) , Label: eFluor 570 , Thermo Fisher Scientific (eBioscience) , Cat: 41-9003-80, 41-9003-82 , Clone: AE1/AE3 , RRID: AB_11218704
2 , Target: E-cadherin , Label: Alexa Fluor 555 , Cell Signaling Technology , Cat: 4295 , Clone: 24E10 , RRID: AB_2728822
2 , Target: H2AX , Label: Alexa Fluor 488 , BioLegend , Cat: 613406 , Clone: 2F3 , RRID: AB_2248011
2 , Target: Histone H3-Lys27-Trimethyl , Label: Alexa Fluor 750 , Cell Signaling Technology , Cat: 98316 , Clone: C36B11 , RRID: AB_2943245
2 , Target: Ki-67 , Label: Alexa Fluor 488 , Cell Signaling Technology , Cat: 11882S , Clone: D3B5 , RRID: AB_2687824
2 , Target: Lysozyme C , Label: Alexa Fluor 790 , Santa Cruz Biotechnology , Cat: sc-518012 AF790 , Clone: E-5 , RRID: AB_2943318
2 , Target: Mast Cell Tryptase , Label: Alexa Fluor 790 , Santa Cruz Biotechnology , Cat: sc-59587 AF790 , Clone: AA1 , RRID: AB_2943323
2 , Target: MITF , Label: Alexa Fluor 488 , Abcam , Cat: ab201675 , Clone: D5 , RRID: AB_2728787
2 , Target: N/A , Label: Alexa Fluor 488 , Invitrogen , Cat: A-11070 , Clone: , RRID: AB_2534114
2 , Target: Nestin , Label: eFluor 570 , Thermo Fisher Scientific , Cat: 41-9843-80 , Clone: 10C2 , RRID: AB_2573652
2 , Target: PD-L1 , Label: Alexa Fluor 647 , Cell Signaling Technology , Cat: 15005 , Clone: E1L3N , RRID: AB_2728832
2 , Target: PD1 , Label: phycoerythrin , Cell Signaling Technology , Cat: 60333S , Clone: D4W2J , RRID: AB_2943233
2 , Target: PDPN , Label: Alexa Fluor 647 , BioLegend , Cat: 916610 , Clone: , RRID: AB_2810816
2 , Target: pMLC2 , Label: Alexa Fluor 488 , Cell Signaling Technology , Cat: 35145BC , Clone: E2J8F , RRID:
2 , Target: PRAME , Label: Alexa Fluor 488 , Cell Signaling Technology , Cat: 39509 , Clone: E7I1B , RRID: AB_2943228
2 , Target: S100B , Label: Alexa Fluor 555 , Abcam , Cat: ab274881 , Clone: EP1576Y , RRID: AB_2890062
2 , Target: SOX10 , Label: Alexa Fluor 647 , Abcam , Cat: ab270151 , Clone: SP267 , RRID: AB_2927700
1&2 , Target: Catenin beta-1 , Label: Alexa Fluor 488 , Cell Signaling Technology , Cat: 2849 , Clone: L54E2 , RRID: AB_10693296

1&2 , Target: CD11b , Label: Alexa Fluor 647 , Abcam , Cat: ab204471 , Clone: EPR1344 , RRID: AB_204471
1&2 , Target: CD11c , Label: Alexa Fluor 555 , Cell Signaling Technology , Cat: 77882BC , Clone: D3V1E , RRID:
1&2 , Target: CD163 , Label: Alexa Fluor 555 , Abcam , Cat: ab281746 , Clone: EPR19518 , RRID: AB_2940922
1&2 , Target: CD20 , Label: Alexa Fluor 488 , eBioscience , Cat: 53-0202-80, 53-0202-82 , Clone: L26 , RRID: AB_10734357
1&2 , Target: CD206 , Label: Alexa Fluor 555 , Cell Signaling Technology , Cat: 48352BC , Clone: E2L9N , RRID:
1&2 , Target: CD31 , Label: Alexa Fluor 647 , Abcam , Cat: ab218582 , Clone: EPR3094 , RRID: AB_2857973
1&2 , Target: CD3E , Label: Alexa Fluor 555 , Cell Signaling Technology , Cat: 57869BC , Clone: D7A6E , RRID:
1&2 , Target: CD4 , Label: Alexa Fluor 488 , R&D Systems , Cat: FAB8165G , Clone: , RRID: AB_2728839
1&2 , Target: CD8a , Label: eFluor 660 , eBioscience , Cat: 50-0008-82 , Clone: AMC908 , RRID: AB_2574149
1&2 , Target: FOXP3 , Label: eFluor 570 , eBioscience , Cat: 41-4777-80, 41-4777-82 , Clone: 236A/E7 , RRID: AB_2573609
1&2 , Target: Granzyme B , Label: Alexa Fluor 647 , Santa Cruz Biotechnology , Cat: sc-8022 AF647 , Clone: 2C5 , RRID: AB_2232723
1&2 , Target: HLA-A and HLA-B , Label: Alexa Fluor 488 , Abcam , Cat: ab198376 , Clone: EPR1394Y , RRID: AB_2943099
1&2 , Target: HLA-DPB1 , Label: Alexa Fluor 647 , abcam , Cat: ab201347 , Clone: EPR11226 , RRID: AB_2861375
1&2 , Target: Hoechst 33342 , Label: , Life Technologies , Cat: H3570 , Clone: , RRID: AB_2651135
1&2 , Target: IRF1 , Label: Alexa Fluor 647 , Cell Signaling Technology , Cat: 14105 , Clone: D5E4 , RRID: AB_2798393
1&2 , Target: LAG3 , Label: Alexa Fluor 555 , Cell Signaling Technology , Cat: 56141BC , Clone: D2G4O™ , RRID: AB_2798739
1&2 , Target: Lamin-A/C , Label: Alexa Fluor 488 , Cell Signaling Technology , Cat: 8617S , Clone: 4C11 , RRID: AB_10997529
1&2 , Target: Lamin-B1 , Label: Alexa Fluor 488 , Abcam , Cat: ab194106 , Clone: EPR8985(B) , RRID: AB_2728786
1&2 , Target: MART-1 , Label: , Abcam , Cat: ab210546 , Clone: EPR20380 , RRID: AB_2889292
1&2 , Target: MX1 , Label: Alexa Fluor 488 , Cell Signaling Technology , Cat: 7937BC , Clone: D3W7I , RRID: AB_2799122
1&2 , Target: Neurofilament L , Label: Alexa Fluor 488 , Cell Signaling Technology , Cat: 8024 , Clone: C28E10 , RRID: AB_10860421
1&2 , Target: PD-1 , Label: Alexa Fluor 647 , Abcam , Cat: ab201825 , Clone: EPR4877(2) , RRID: AB_2728811
1&2 , Target: PMEL , Label: phycoerythrin , Abcam , Cat: ab246731 , Clone: EP4863(2) , RRID: AB_2890052
1&2 , Target: S100 alpha , Label: Alexa Fluor 488 , Abcam , Cat: ab207367 , Clone: EPR5251 , RRID: AB_2728788
1&2 , Target: SOX9 , Label: Alexa Fluor 488 , Abcam , Cat: ab196450 , Clone: EPR14335 , RRID: AB_2665383
1&2 , Target: Tubulin beta chain , Label: Alexa Fluor 555 , Cell Signaling Technology , Cat: 2116S , Clone: 9F3 , RRID: AB_10695881
1&2 , Target: Vimentin , Label: Alexa Fluor 750 , Cell Signaling Technology , Cat: 69227BC , Clone: D21H3 , RRID:

Validation

Each antibody was tested on clinical discard and tonsil samples to verify expected and unexpected staining patterns in positive and negative controls. Staining patterns were visually certified by Board Certified Pathologists.

Plants

Seed stocks

N/A

Novel plant genotypes

N/A

Authentication

N/A
